# Plasma metabolome and cognitive skills in Down syndrome

**DOI:** 10.1038/s41598-020-67195-z

**Published:** 2020-06-26

**Authors:** Francesca Antonaros, Veronica Ghini, Francesca Pulina, Giuseppe Ramacieri, Elena Cicchini, Elisa Mannini, Anna Martelli, Agnese Feliciello, Silvia Lanfranchi, Sara Onnivello, Renzo Vianello, Chiara Locatelli, Guido Cocchi, Maria Chiara Pelleri, Lorenza Vitale, Pierluigi Strippoli, Claudio Luchinat, Paola Turano, Allison Piovesan, Maria Caracausi

**Affiliations:** 1 Department of Experimental, Diagnostic and Specialty Medicine (DIMES), Unit of Histology, Embryology and Applied Biology, University of Bologna, Via Belmeloro 8, 40126 Bologna, BO Italy; 2 CIRMMP, Consorzio Interuniversitario Risonanze Magnetiche Metallo Proteine, via Luigi Sacconi 6, 50019 Sesto Fiorentino, Florence, FI Italy; 3 Department of Developmental Psychology and Socialization, University of Padova, Via Venezia 8, 35131 Padova, PD Italy; 4 Neonatology Unit, St. Orsola-Malpighi Polyclinic, Department of Medical and Surgical Sciences (DIMEC), University of Bologna, Via Massarenti 9, 40138 Bologna, BO Italy; 5 Neonatology Unit, St. Orsola-Malpighi Polyclinic, Via Massarenti 9, 40138 Bologna, BO Italy; 6 CERM, Center of Magnetic Resonance and Department of Chemistry, University of Florence, via Luigi Sacconi 6, 50019 Sesto Fiorentino, Florence, Italy

**Keywords:** Metabolomics, Genomics, Medical genetics, Neurodevelopmental disorders, Biochemistry, Chemistry

## Abstract

Trisomy 21 (Down syndrome, DS) is the main human genetic cause of intellectual disability (ID). Lejeune hypothesized that DS could be considered a metabolic disease, and we found that subjects with DS have a specific plasma and urinary metabolomic profile. In this work we confirmed the alteration of mitochondrial metabolism in DS and also investigated if metabolite levels are related to cognitive aspects of DS. We analyzed the metabolomic profiles of plasma samples from 129 subjects with DS and 46 healthy control (CTRL) subjects by ^1^H Nuclear Magnetic Resonance (NMR). Multivariate analysis of the NMR metabolomic profiles showed a clear discrimination (up to 94% accuracy) between the two groups. The univariate analysis revealed a significant alteration in 7 metabolites out of 28 assigned unambiguously. Correlations among the metabolite levels in DS and CTRL groups were separately investigated and statistically significant relationships appeared. On the contrary, statistically significant correlations among the NMR-detectable part of DS plasma metabolome and the different intelligence quotient ranges obtained by Griffiths-III or WPPSI-III tests were not found. Even if metabolic imbalance provides a clear discrimination between DS and CTRL groups, it appears that the investigated metabolomic profiles cannot be associated with the degree of ID.

## Introduction

Down syndrome (DS) is the most common chromosomal disorder described in humans^1^ and affects approximately 1 in every 1000–1100 newborns worldwide^2^. It is caused by the presence of human chromosome 21 in three copies in the cells of affected subjects. The main features of DS consist of intellectual disability (ID), cardiovascular defects and craniofacial dysmorphisms^3^.

After a systematic reanalysis of all described partial trisomy 21 cases, Pelleri *et al*.^4^ identified a highly restricted Down syndrome critical region (HR-DSCR) on human chromosome 21. This region is 34 kb long and is located on the distal 21q22.13. It is duplicated in all DS subjects while it is not duplicated in subjects without a diagnosis of DS^5^. To date there is no information about the role of this region (HR-DSCR), which does not include known genes but which is part of a very long intron of the isoform 2 of the *KCNJ6* gene (https://www.ncbi.nlm.nih.gov/gene/3763)^5^.

Recently, Caracausi *et al*.^6^ conducted an analysis of the ^1^H Nuclear Magnetic Resonance (NMR)-detectable part of the metabolome in plasma and urine samples of 67 DS subjects and 29 healthy controls (CTRLs) for the first time. They found that DS samples have an altered metabolomic profile, in particular significantly modified levels of several metabolites were observed and attributed to mitochondrial dysmetabolism.

Lejeune had already hypothesized that DS can be considered a metabolic disease^7^, suggesting that a “blocked” mechanism might determine the level of ID severity, and that specific molecular protagonists of this complex mechanism might be identified.

Finding the possible relationship between metabolomic profile and ID in DS might be of immense value. Searching for the main altered gene products instead for the gene defects may change the point of view of the possible treatment of this condition.

ID is considered to be a significant limitation in both intellectual functioning and adaptive behavior. The mental development of children with DS shows a deceleration during the developmental period^8^ and their final intelligence quotient (IQ) ranges between 35 and 70, with a mean value of 50^9,10^. However, since IQ is calculated with respect to the distribution of typically developing children of the same chronological age, IQ tends to decrease with age as an effect of the developmental deceleration characteristic of this syndrome. Moreover a high degree of interindividual variability has been demonstrated^11^.

Previous works indicated that DS is associated with a specific cognitive phenotype, characterized by impairments in speech and language^12^, with greater difficulties in expressive language than in receptive language, working memory^13^, as well as in executive functions^13,14^. Considering motor skills, subjects with DS have fine motor problems due to specific visuo-motor integration and eye-hand coordination problems, combined with slow movement^15^. On the other hand, non-verbal skills are less severely affected, though recent studies have shown a variable picture depending on which aspect of visuospatial cognition is considered^16^. Another area of relative strength is social functioning^17^.

In this work we performed the metabolomic analysis of plasma samples from a higher, almost doubled, number of subjects than in the previous work^6^. In addition, whenever possible, the cognitive data of DS subjects were collected, and statistical correlations between the metabolomic profiles and cognitive aspects were carried out. The aim of the present work was to confirm the previously observed changes in the metabolome, to identify if the metabolic imbalance makes a clear discrimination between DS and CTRL groups and to verify if specific metabolomic profiles can be associated with the degree of ID.

## Results

### Study design

Metabolomic data were obtained from a total of 129 subjects with DS (mean age ± standard deviation (SD) = 11.23 ± 6.64 years) and 46 CTRL subjects (mean age ± SD = 15.18 ± 7.99 years) chosen among DS siblings. Sex distribution was 51 female (F) and 78 male (M) among DS and 22 F and 24 M among CTRLs (Table 1). This sample size allowed us to obtain a statistical power of 0.92 (software G*Power, estimation for Wilcoxon-Mann-Whitney test, two tails, effect size d = 0.6, α = 0.05). The main features of the subjects enrolled in this study are described in Table 1. Data concerning age, IQ and medications at the time of the survey are reported in the Supplementary Dataset S1.

**Table 1:** Number of Down syndrome (DS) and healthy control (CTRL) subjects.

Patients	Total	Male	Female	Fasting	Non-Fasting
DS	129	78	51	76	53
CTRL	46	24	22	35	11

As explained in our previous work^6^, because of the pediatric age of most of the subjects, it was not always possible to collect samples at a fasting state. Thus, to avoid that non-fasting conditions could alter the results, we performed multivariate and univariate analyses for two groups of subjects: the “all” group including fasting and non-fasting subjects, and the “fasting” group (76 DS and 35 CTRL).

With the aim of minimizing healthcare-induced stress, the metabolomic analyses were conducted on EDTA-plasma samples (as in the previous study^6^). This sample type is indeed suitable for multiple *in vitro* testing using different techniques, including the analysis of circulating microRNA^18^.

### Plasma metabolome analysis

**Figure 1:**
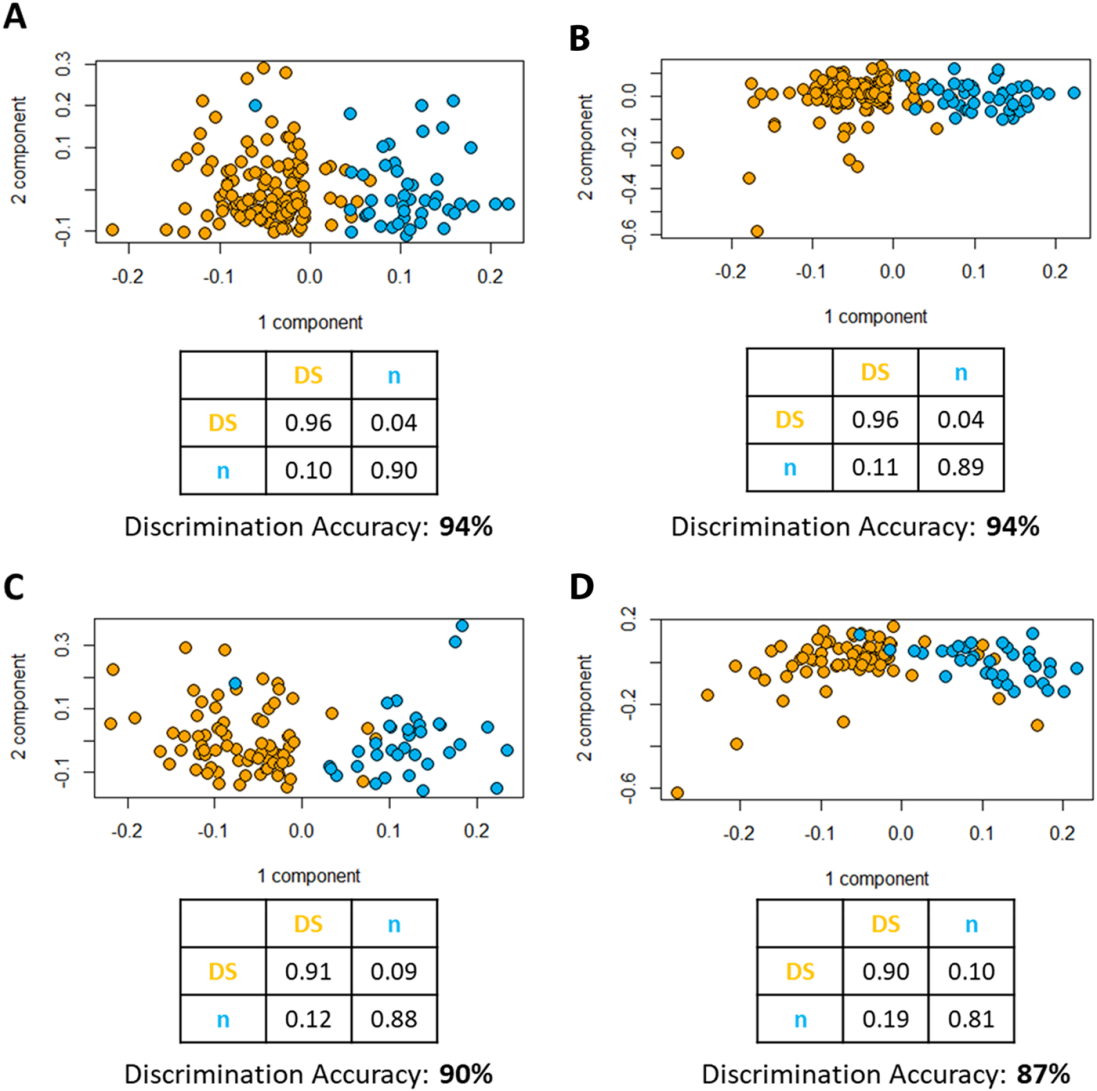
PLS-CA analysis of all the plasma samples: (**A**) CPMG and **(B)** NOESY spectra. Score plot, each dot represents a different plasma sample. Orange dots: Down syndrome samples (DS, n = 129); blue dots: healthy controls (CTRL, n = 46). PLS-CA analysis of the fasting plasma samples: **(C)** CPMG and **(D)** NOESY spectra. Score plot, each dot represents a different plasma sample. Orange dots: Down syndrome samples (DS, n = 76); blue dots: healthy controls (CTRL, n = 35).

Partial Least Squares-Canonical analysis (PLS-CA) of all plasma samples discriminated DS and CTRL groups with an accuracy of 94% in both CPMG (Carr-Purcell-Meiboom-Gill) and NOESY (Nuclear Overhauser Effect Spectroscopy) spectra (Fig. 1A,B, respectively). Under fasting conditions, the discrimination accuracies were 90% in CPMG spectra (Fig. 1C) and 87% in NOESY spectra (Fig. 1D).

A random selection of 46 DS and 46 CTRL samples derived from all subjects, repeated 500 times, highlighted a discrimination between DS and CTRL groups with an average accuracy of 87% (p-value < 0.01) in CPMG spectra and 88% (p-value < 0.01) in NOESY spectra, Supplementary Figures S1A and S1B. Considering only fasting subjects, a casual selection of 35 DS and 35 CTRL samples, repeated 500 times, showed a discrimination between DS and CTRL groups with a mean accuracy of 87% in CPMG spectra and 86% in NOESY spectra (p-value < 0.01 in both analyses), Supplementary Figures S1C and S1D.

**Figure 2:**
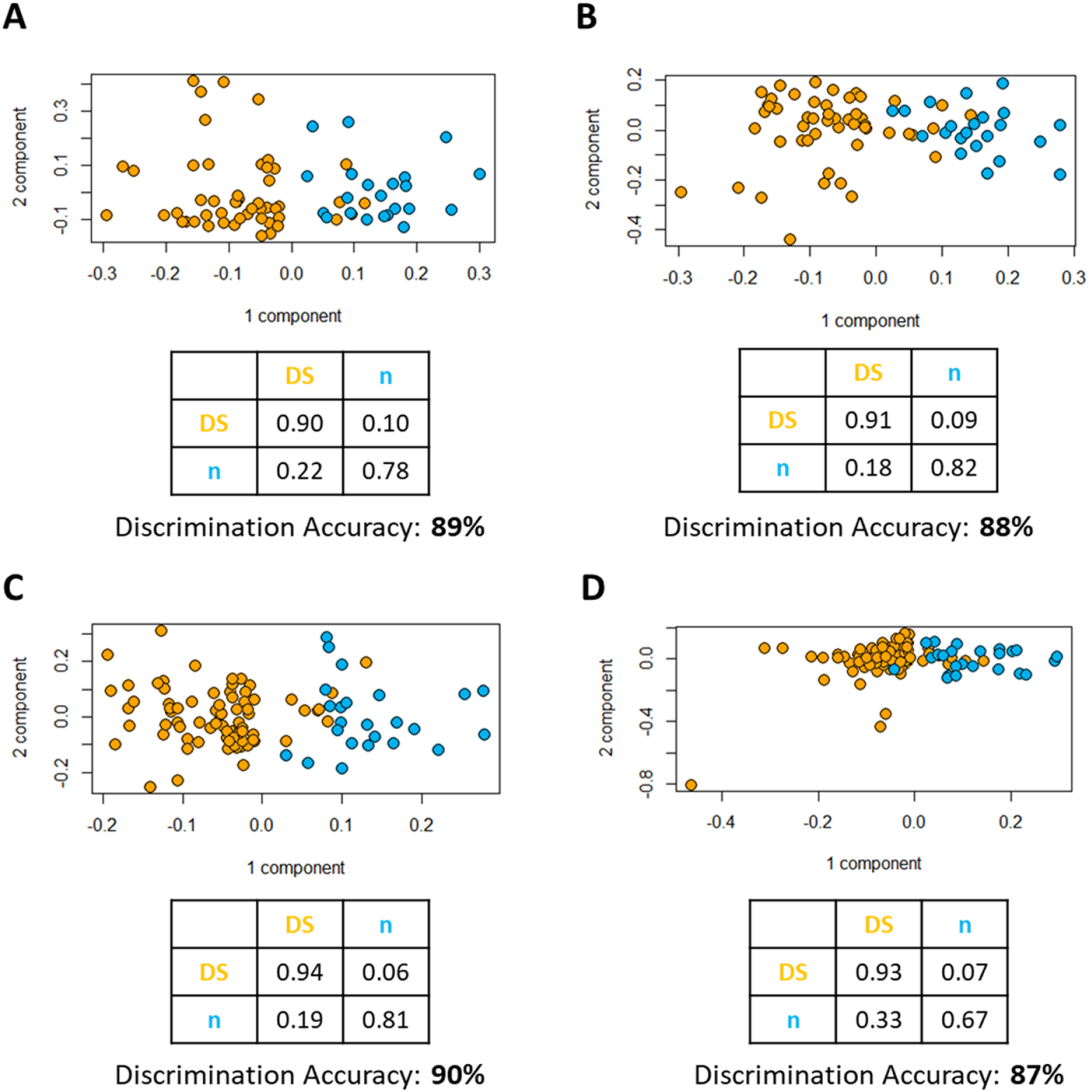
PLS-CA analysis of all the plasma samples from female subjects: **(A)** CPMG and **(B)** NOESY spectra. Score plot, each dot represents a different plasma sample. Orange dots: Down syndrome samples (DS, n = 51); blue dots: healthy controls (CTRL, n = 22). PLS-CA analysis of all the plasma samples from male subjects: **(C)** CPMG and **(D)** NOESY spectra. Score plot, each dot represents a different plasma sample. Orange dots: Down syndrome samples (DS, n = 78); blue dots: healthy controls (CTRL, n = 24).

The same analyses were also performed with samples grouped by sex. In all-female samples, PLS-CA discriminated DS from CTRL groups with an accuracy of 89% (p-value < 0.01) in CPMG spectra (Fig. 2A), while in NOESY spectra (Fig. 2B) the discrimination accuracy was 88% (p-value < 0.01). Discrimination accuracy in all-male groups was 90% (p-value < 0.01) in CPMG spectra (Fig. 2C) and 87% (p-value < 0.01) in NOESY spectra (Fig. 2D). Considering only fasting female subjects, the discrimination accuracies were 84% and 85% (p-value < 0.01) in CPMG and NOESY spectra, respectively. Considering only fasting male subjects, the discrimination accuracy was 86% (p-value < 0.01) in both CPMG and NOESY spectra.

Among female samples, a random selection of 22 DS and 22 CTRLs, repeated 500 times, gave a discrimination between DS and CTRL groups with a mean accuracy of 82% in CPMG spectra (p-value < 0.01) and 85% in NOESY spectra (p-value < 0.01), Supplementary Figure S2A and S2B. Among male samples, a random selection of 24 DS and 24 CTRL, repeated 500 times, gave a discrimination between DS and CTRL groups with a mean accuracy of 80% in CPMG spectra (p-value < 0.01) and 76% in NOESY spectra (p-value < 0.01), Supplementary Figure S2C and S2D.

The signals of 28 metabolites were unambiguously assigned in all the NMR spectra (Supplementary Dataset S1). Their levels, together with the levels of 3 unknown signals (unk1, unk2 and unk3), were analyzed through univariate statistical analysis (Tables 2 and 3). Considering the samples from all subjects, independently from their fasting state, acetate, pyruvate, acetone, creatine, formate, acetoacetate, unk3 and succinate showed significantly increased levels in DS, with a DS/CTRL ratio > 1 whereas unk1, tyrosine, histidine and threonine showed significantly reduced levels in DS, with a DS/CTRL ratio < 1 (Table 2). Taking into account only fasting samples, the same metabolites were also found to be significantly increased or decreased in DS, with the exception of acetone, threonine and unk1, which resulted not significantly different in the two groups, and of methionine, which increases significantly in DS plasma (Table 3).

**Table 2:** Univariate statistical analysis of all plasma samples (Down syndrome (DS), n = 129; healthy control (CTRL), n = 46).

Metabolites	DS (median)	CTRL (median)	DS/CTRL median	p-value	p-value after FDR Correction
Leucine	0.00344507	0.003570211	0.96	0.1372	0.2658
Isoleucine	0.00040417	0.000429518	0.94	0.0794	0.1797
Valine	0.00352026	0.003575671	0.98	0.2910	0.4748
Alanine	0.00475446	0.004815092	0.99	0.4072	0.5460
**Acetate**	0.00089010	0.000736686	1.21	**0.0086**	**0.0443**
**Pyruvate**	0.00222810	0.002017161	1.10	**0.0110**	**0.0485**
**Acetone**	0.00057313	0.000491021	1.17	**0.0461**	0.1190
Glutamine	0.00628499	0.006405399	0.98	0.8455	0.8455
Citrate	0.00041200	0.000393842	1.05	0.3132	0.4855
Glycine	0.00210159	0.002229647	0.94	0.4507	0.5589
**Creatine**	0.00115954	0.001007514	1.15	**0.0002**	**0.0018**
Creatinine	0.00041121	0.000458047	0.90	0.0812	0.1797
Lactate	0.00396610	0.003596582	1.10	0.1983	0.3616
Glucose	0.02785484	0.027973926	1.00	0.6730	0.7194
Mannose	7.778E-05	8.34062E-05	0.93	0.6267	0.7011
**Tyrosine**	0.00059000	0.000668668	0.88	**0.0184**	0.0651
**Histidine**	0.00031190	0.000349493	0.89	**0.0189**	0.0651
Phenylalanine	0.00035077	0.000352009	1.00	0.4818	0.5745
**Formate**	0.00020871	0.000183081	1.14	**0.0085**	**0.0443**
Fumarate	0.00001230	1.15222E-05	1.07	0.4227	0.5460
**Threonine**	0.00019038	0.000220489	0.86	**0.0315**	0.0880
Lysine	0.00047591	0.000466559	1.02	0.8111	0.8382
**Acetatoacetate**	0.00027010	0.000167440	1.61*	**0.0241**	0.0746
Methionine	0.00079102	0.000732161	1.08	0.0924	0.1909
**unk1**	0.00006890	8.40005E-05	0.82	**0.0039**	**0.0305**
unk2	5.3121E-05	4.60515E-05	1.15	0.2154	0.3709
**unk3**	0.00099389	0.000691254	1.44*	**0.0000001**	**0.0000009**
3-hydroxybutyrate	0.00041516	0.000380792	1.09	0.4091	0.5460
**Succinate**	0.00006360	4.63183E-05	1.37*	**0.000001**	**0.000009**
**2-hydroxybutyrate**	0.00010237	7.38223E-05	1.39*	0.6333	0.7013
Proline	0.00025393	0.000246843	1.03	0.4188	0.5460

The table contains the list of metabolites analyzed with NMR. We reported both p-value of the univariate Wilcoxon test and p-value after FDR correction for each metabolite. Metabolites that show significant concentration differences in the two groups (p-value < 0.05) and/or show values in the interval next to 3:2 are reported in bold. *Values in the interval next to 3:2 (range 1.3–1.7).

**Table 3:** Univariate statistical analysis of fasting samples (Down syndrome (DS), n = 76; healthy control (CTRL), n = 35).

Metabolites	DS (median)	CTRL (median)	DS/CTRL median	p-value	p-value after FDR Correction
Leucine	0.00361293	0.003694505	0.98	0.3591	0.4840
Isoleucine	0.00042202	0.000475091	0.89	0.1063	0.2059
Valine	0.00374662	0.003824309	0.98	0.5700	0.6796
Alanine	0.00483927	0.004903123	0.99	0.5277	0.6544
**Acetate**	0.00090934	0.000736206	1.24	**0.0015**	**0.0137**
**Pyruvate**	0.00226253	0.002109906	1.07	**0.0085**	0.0528
Acetone	0.00057523	0.000517008	1.11	0.1857	0.3199
Glutamine	0.00642759	0.006454857	1.00	0.8415	0.8696
Citrate	0.00042516	0.000385006	1.10	0.0996	0.2058
Glycine	0.00215948	0.002230915	0.97	0.6409	0.7358
**Creatine**	0.00114727	0.001000137	1.15	**0.0018**	**0.0137**
Creatinine	0.00045933	0.000489828	0.94	0.7342	0.7848
Lactate	0.00413212	0.003602912	1.15	0.0495	0.1395
Glucose	0.02830345	0.027789163	1.02	0.3054	0.4303
Mannose	0.00008250	8.55137E-05	0.96	0.6963	0.7709
**Tyrosine**	0.00060915	0.000681629	0.89	**0.0166**	0.0828
**Histidine**	0.00034121	0.000361783	0.94	**0.0307**	0.1030
Phenylalanine	0.00035893	0.000336898	1.07	0.4111	0.5311
**Formate**	0.00020937	0.00018	1.16	**0.0187**	0.0828
Fumarate	0.00001415	1.25068E-05	1.13	0.2377	0.3879
Threonine	0.00020752	0.000231226	0.90	0.0932	0.2058
Lysine	0.00049623	0.000488073	1.02	0.9873	0.9873
**Acetatoacetate**	0.00026706	0.000167242	1.60*	**0.0265**	0.1029
**Methionine**	0.00082180	0.000730695	1.12	**0.0332**	0.1030
unk1	0.00008045	8.6702E-05	0.93	0.0920	0.2058
unk2	0.00005625	4.58131E-05	1.23	0.0957	0.2058
**unk3**	0.00104660	0.000699813	1.50*	**0.0000016**	**0.000025**
3-hydroxybutyrate	0.00042926	0.000398342	1.08	0.2994	0.4303
**Succinate**	7.3119E-05	4.65665E-05	1.57*	**0.0000001**	**0.0000035**
**2-hydroxybutyrate**	0.00011189	7.75603E-05	1.44*	0.1753	0.3196
Proline	0.00025412	0.000230208	1.10	0.2878	0.4303

The table contains the list of metabolites analyzed with NMR from samples taken from fasting patient. We reported both p-value of the univariate Wilcoxon test and p-value after FDR correction for each metabolite. Metabolites that show significant concentration differences in the two groups (p-value < 0.05) and/or show values in the interval next to 3:2 are reported in bold. *Values in the interval next to 3:2 (range 1.3–1.7).

### Correlations between metabolites in DS and control groups

**Figure 3:**
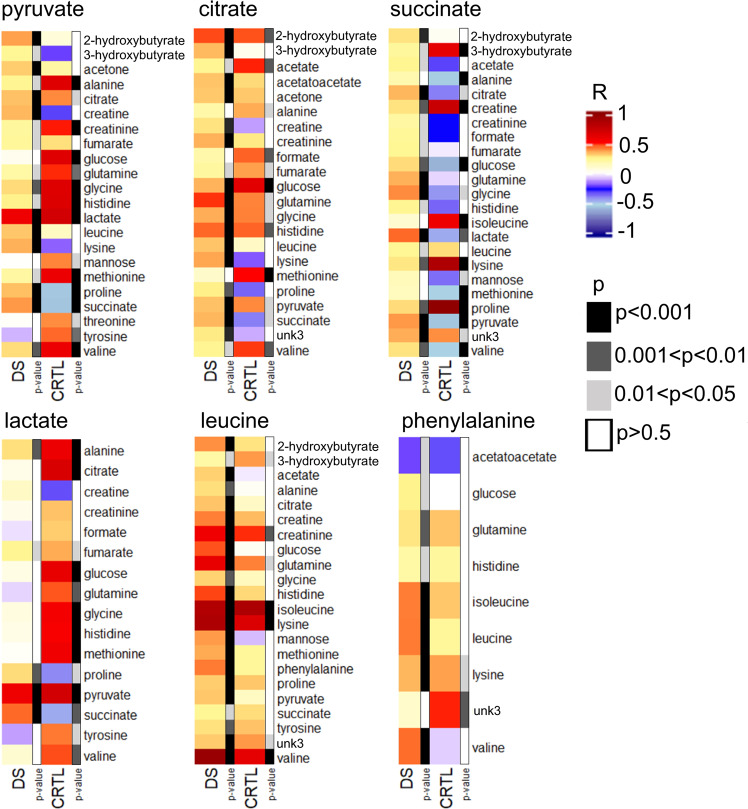
Statistically significant correlations between the levels of some of the altered metabolites and all other detected metabolites. Heat maps of the correlations (r) of representative metabolites in both Down syndrome (DS) and healthy control (CTRL) samples, separately. The p-value (after FDR correction) of each correlation is also reported.

We compared the levels of each metabolite with those of all the others performing a series of correlation analyses in both DS and CTRL samples, separately. Partial correlations including “chronological age” as covariate were conducted (see “Statistical analysis” paragraph in the “Materials and methods” section). We considered only correlations with p-value after FDR (False Discovery Rate)  < 0.05 and with a correlation coefficient r > 0.4 or < −0.4 to be statistically significant. We clustered the results into 7 main groups of metabolites whose levels have statistically significant correlations: the Krebs cycle metabolites (Supplementary Table 1); formate (Supplementary Table 2); ketone bodies (Supplementary Table 3); lactate, glucose and mannose (Supplementary Table 4); branched-chain amino acids (BCAA) (Supplementary Table 5); creatine and creatinine (Supplementary Table 6); amino acids (Supplementary Table 7). Interestingly, some correlations found in CTRL samples had no correspondence in DS samples and *vice versa* (Supplementary Tables 1, 2, 3, 4, 5, 6, 7; Fig. 3).

### Correlations between metabolome and cognitive skills

We investigated if the NMR-detectable part of the plasma metabolome of subjects with DS contains signatures of the different IQ scores.

39 subjects with DS were evaluated through the WPPSI-III test (Supplementary Dataset S1). We created two main groups of DS patients on the basis of their IQ scores: one group included all DS subjects with an IQ > 40 (n = 13, mean age=125.69 months) and the other one included all DS subjects with an IQ ≤ 40 (n = 26, mean age=141.38 months). The difference between the two mean ages was not statistically significant by unpaired t-test (t = −1.694; p = 0.099). Using the PLS-CA model we obtained a discrimination accuracy of 74.5% between the plasma metabolomic profiles of the two groups, i.e. DS subjects with IQ > 40 vs. IQ ≤ 40 (Supplementary Figure S3). Additionally, we were not able to identify any metabolites with statistically significant concentration differences between the two groups.

22 subjects with DS were evaluated using the Griffiths-III test (Supplementary Dataset S1). Using the same statistical approach specified for the WPPSI-III test, we obtained a discrimination accuracy of 74.3% between the metabolomic profiles of DS subjects with IQ > 40 (n = 17, mean age=55.94 months) and those one with IQ ≤ 40 (n = 5, mean age=66.4 months). Again, the difference between the two mean ages was not statistically significant by unpaired t-test (t = −1.997; p = 0.06). Also in this case we were not able to identify any metabolites with statistically significant concentration differences (Supplementary Figure S4).

Furthermore, we performed a series of correlation analyses in order to investigate correlations between each metabolite level and the age equivalent (AE) scores collected from Griffiths-III and WPPSI-III tests from DS patients (see AE scores in the Supplementary Dataset S1 and Supplementary Tables 8 and 9). Partial correlations including “chronological age” as covariate were conducted (see “Statistical analysis” paragraph in the “Materials and methods” section). We were unable to obtain any statistically significant correlations considering p < 0.01 and r < −0.4 or r > 0.4 (Supplementary Tables 8 and 9).

## Discussion

The aim of this study was to establish whether an alteration in metabolite levels may have an important role in DS. For this reason, we analyzed NMR spectra of plasma samples from patients with DS and their siblings, used as CTRL group, thus significantly increasing the number of subjects with respect to our previous work^6^ (from 67 to 129 DS subjects and from 29 to 46 CTRLs). First of all, we confirmed the presence of significantly different plasma metabolomic profiles of DS and CTRL groups and identified the metabolites with significantly different concentrations in the two groups. Then, we searched for correlations among metabolite levels in DS and CTRL samples separately and correlations between metabolite levels and cognitive data of DS subjects. Finally, we investigated whether the characteristic plasma metabolomic profile of DS subjects contains signatures of different grades of ID.

The discrimination accuracy between DS and CTRL groups is higher than in our previous analysis^6^, reaching 94% both with CPMG and NOESY spectra (Fig. 1A,B). If we only consider fasting subjects, the discrimination accuracy only slightly decreases to 90% with CPMG spectra and 87% with NOESY spectra (Fig. 1C,D). The decrease can be explained considering the reduction in the sample size using only fasting samples. Sex is not a confounding factor; in fact, our results show similar discrimination accuracy between DS and CTRL groups when considering females and males separately in both CPMG and NOESY spectra (again, a slight decrease in discrimination accuracy with respect to the complete group can be attributed to the reduction in the numbers of individuals). This is a further confirmation of our previous work^6^, where we demonstrated that both sex and age were not confounding factors for the discrimination between DS and CTRL metabolomic profiles.

The analysis of plasma samples provided the concentrations of 28 different metabolites involved in multiple metabolic pathways.

The univariate analysis confirmed the significant alterations of the levels of metabolites involved in processes related to mitochondrial metabolism in DS highlighted by Caracausi *et al*.^6^; the DS/CTRL ratio allowed us to understand when the metabolite level increases (DS/CTRL > 1) or decreases (DS/CTRL < 1) in DS vs CTRL samples. The analysis showed significantly increased concentrations (p-value after FDR correction < 0.05) of the following metabolites: acetate; pyruvate; succinate; formate and creatine. All of them are involved in energy production. Acetate is fundamental in supporting acetyl-coenzyme A metabolism and thus Krebs cycle progression and lipogenesis^19^. Pyruvate and succinate are produced before and during the Krebs cycle^20^. Formate is known to be necessary for the formylation of mitochondrial tRNAs^21^ and for the formylation of tetrahydrofolate during the folic acid cycle and the purine synthesis pathway. Finally creatine is used by muscles for the production of ATP^22^. Previous works demonstrated that measurable enzymes whose genes are located on Hsa21 adhere to the 3:2 overexpression model expected in trisomy 21^23–27^. Although the dependence of the levels of the metabolites on the enzyme concentration is complex and not easily interpretable, we point out that a ratio DS/CRTL near to 3:2 (1.5) or 2:3 (0.67) is observed for a certain set of molecules. In this framework, interesting data are the significant alterations of succinate in DS vs CTRL. Succinate levels increase in DS samples (p-value after FDR correction <0.0001 in both “all samples” and “fasting samples” groups) with a DS/CTRL ratio close to 3:2 (1.37 in “all samples” and 1.57 in “fasting samples” groups).

The alterations of succinate metabolism may play a role in the development of ID, but to date there is no strong evidence. However, it is already known that some enzymes which metabolize succinate are involved in developmental delay and brain injuries. In particular, the succinic semialdehyde dehydrogenase (SSADH) deficiency, caused by a mutation of the *ALDH5A1* gene (6p22.3), determines an increase of γ-aminobutyric acid (GABA) and γ-hydroxybutyric acid levels. These alterations cause developmental delay, hypotonia, hyporeflexia, ataxia, neuropsychiatric problems, and epilepsy^28^.

Furthermore, alterations of activities for the Krebs cycle enzymes were also observed in some brain disorders such as Alzheimer’s disease^29^ and Huntington’s disease^30^. In both diseases, the increase of succinate dehydrogenase and malate dehydrogenase and the decrease of the pyruvate dehydrogenase complex, isocitrate dehydrogenase, and the alpha-ketoglutarate dehydrogenase complex were observed, suggesting that a mitochondrial alteration occurs. These results also suggest that measures to improve tricarboxylic acid cycle metabolism might lessen the effects of the diseases^29,30^.

In order to obtain more information about the alteration of the metabolic pathways in DS we compared the correlations (corrected by age) among the levels of metabolites in DS subjects and CTRL groups, separately.

Importantly, we observed that some correlations are strong/moderate (r > 0.4 or r < −0.4) and significant (p < 0.05) in CTRL samples but lose their significance or weaken their correlation in DS samples. These results are referred to metabolites involved in the Krebs cycle (pyruvate, citrate and succinate), formate and lactate and amino acids like alanine, threonine and tyrosine (Supplementary Table 1, 2, 4, 7; Fig. 3). On the contrary, some statistically significant correlations are found only in DS samples, like associations between phenylalanine and branched amino acids (Supplementary Table 7; Fig. 3). Moreover, the loss of correlations among strictly related metabolites (from a biochemical point of view) and the appearance of new correlation patterns in DS confirmed the dysregulation of some metabolic pathways. Accordingly, the inverse correlation between lactate and succinate characteristic of CTRL samples becomes direct in DS samples (Supplementary Table 1 and 4; Fig. 3).

These findings support the hypothesis of an altered metabolism in DS, strengthening the idea of the involvement of the Krebs cycle and of a few amino acids like leucine, phenylalanine, tyrosine and alanine. Leucine and phenylalanine are both involved in brain metabolism: the role of leucine as a nitrogen donor in the glutamine/glycine pathway may influence the function of some neurotransmitters^31^, while modifications in the phenylalanine pathway can alter dopamine levels^32^.

Even if metabolic imbalance clearly discriminates between DS and CTRL groups, it appears that specific metabolomic profiles cannot be associated with the degree of ID, as shown by both the low discrimination accuracies obtained when comparing the metabolomic profiles of DS patients with different IQ disability and the lack of correlation between the levels of the investigated metabolites and the scores obtained by cognitive tests (Supplementary Tables 8 and 9). It would be interesting to test if strengthening the statistical power of the models by increasing the number of patients in each range of ID could reveal such a correlation, as it would be of immense value in identifying critical points of metabolism as treatment targets. An alternative or complementary explanation for this result might be that, in accordance with the neuroconstructivist perspective on development, the effect of metabolome on patient’s skills might be mediated by environmental factors (such as family environment, school, early intervention, etc.), so further study is required here.

In addition, it is possible that metabolites critically altered in DS are not included among those we have investigated (i.e., fall below the NMR detection limit). This hypothesis should be further investigated by studying a larger number of metabolites using complementary analytical platforms with a better sensitivity with respect to NMR, like mass spectrometry^33,34^. By increasing the number of measured metabolites, extended metabolic network models could be developed and might provide deeper insight in the biochemical origin of cognitive impairment^35,36^.

## Materials and methods

### Ethics Statement

The study was approved by the independent Ethics Committee of the University Hospital St. Orsola-Malpighi Polyclinic, Bologna, Italy (study number: 39/2013/U/Tess). Informed written consent was obtained from all participants or their legal guardians. The patients, if over 18, or their legal guardians, signed an informed consent form for the collection of blood and clinical data before taking part in the study. All procedures were carried out in accordance with the Ethical Principles for Medical Research Involving Human Subjects of the Helsinki Declaration.

### Case selection

All subjects were admitted to the Day Hospital of the Neonatology Unit, Sant’Orsola-Malpighi Polyclinic, Bologna, and this study was proposed in the context of the yearly routine follow-up provided for DS. Inclusion criteria for subjects with DS were diagnosis of DS with homogeneous or mosaic trisomy 21 and minimum age of 2 years.

A total of 175 subjects, between 3 and 37 years old, participated in the study including 129 patients with DS and 46 healthy CTRLs chosen among DS siblings and with no evidence of abnormal karyotype. 26 DS subjects had at least one sibling enrolled in the study. Blood samples, as well as clinical data, were collected during the follow-up visit.

For every collected sample, parents filled out a form with information about current fasting state, last meal, consumed medications.

### Plasma sample preparation

Plasma samples were collected from all subjects enrolled in the study, according to standard procedures^37–39^.

Blood samples were collected in EDTA-coated blood collection tubes and stored at room temperature. They were treated within two hours of blood collection and every delayed treatment of the sample was recorded.

The samples were transferred to a new tube and centrifuged at 1,200 g for 10 min to separate corpuscular fraction from plasma. The plasma fraction was isolated and centrifuged for a second time at 800 g for 30 min. The supernatant was transferred to new tubes avoiding contact with pellets or with the bottom of the tube and then divided in aliquots of 400 μL. All plasma samples were rapidly stored in a − 80 °C freezer.

To avoid contamination all procedures were conducted carefully. The plasma samples were excluded from the analyses when the treatment of blood samples occurred more than two hours from their collection, or when evident contamination by residual erythrocytes at the end of the treatments occurred. Any anomalies like different plasma color or precipitates after centrifugation were noted and considered in further analysis.

### NMR sample preparation, spectra processing and spectral analysis

NMR samples were prepared according to standard procedures^6,40,41^.

NMR spectra for all samples were acquired using a Bruker 600 MHz spectrometer (Bruker BioSpin) operating at 600.13 MHz proton Larmor frequency and equipped with a 5 mm PATXI ^1^H-^13^C-^15^N and ^2^H-decoupling probe including a z-axis gradient coil, an automatic tuning-matching (ATM) and an automatic and refrigerate sample changer (SampleJet, Bruker BioSpin). A BTO 2000 thermocouple served for temperature stabilization at the level of approximately 0.1 K of the sample. Before measurement, samples were kept inside the NMR probe head for 5 minutes for temperature equilibration at 310 K.

For each plasma sample, two monodimensional ^1^H-NMR spectra were acquired with water peak suppression and different pulse sequences that allowed the selective observation of different molecular components. The spectra were: 1) a standard NOESY^42^ using 32 scans, 98,304 data points, a spectral width of 18,028 Hz, an acquisition time of 2.7 s, a relaxation delay of 4 s and a mixing time of 0.1 s. This type of spectrum is made up of signals arising from low molecular weight molecules (metabolites) and signals arising from macromolecules such as lipoproteins and lipids; 2) a standard CPMG (Purcell) using 32 scans, 73,728 data points, a spectral width of 12,019 Hz and a relaxation delay of 4 s. This type of spectrum contains only the signals arising from low molecular weight molecules (metabolites).

Before applying Fourier transform, free induction decays were multiplied by an exponential function equivalent to a 0.3 Hz line-broadening factor. Transformed spectra were automatically corrected for phase and baseline distortions and calibrated to the glucose doubled at δ 5.24 ppm, using TopSpin 3.5 (Bruker BioSpin)^6,40,41^.

### Cognitive data collection

Cognitive data were collected and processed for a total of 61 DS children/adolescents from 3 to 16 years old. Following an increasingly widespread procedure in the field of intellectual disability^43^, the cognitive level was assessed through tests for expected mental age rather than for chronological age. This approach provides a more sensitive measure that avoids floor effects.

Children from 3 to 6 years and 11 months were assessed using the Griffiths-III scale^44^, a play-oriented developmental test. Considering the DS cognitive profile, two scales were used for the purpose of this study: foundation of learning (scale A), which assesses different aspects of thinking; and language and communication (scale B), which measures overall language development, including expressive language, receptive language, and to a lesser extent, the use of language to communicate socially.

Children/adolescents from 7 to 16 years old were assessed using the WPPSI-III scale^45^ which consists of different subtests summarized in three principal indexes: Verbal, Non Verbal and Total.

For both tests, raw scores were registered and later converted into AE scores. However, since the AE scores do not take into account the subject’s age, every statistical analysis involving AE values has been corrected for chronological age.

Moreover, an IQ score was calculated as the ratio of the subject’s AE to his/her chronological age, multiplied by 100.

### Statistical analysis

Multivariate analysis of the NMR data (aimed at analyzing the spectra as a whole) was performed on binned spectra. Each spectrum in the 10.00–0.2 ppm region was segmented into 0.02 ppm chemical shift bins and the corresponding spectral areas were integrated using the AMIX software (Bruker BioSpin). The presence of EDTA as anticoagulant gives rise to few NMR signals that are very intense and whose concentration levels can be slightly different among samples. Thus, together with the spectral region containing water signals (region:4.40–5.00 ppm), spectral regions including EDTA signals (regions: 2.53–2.60, 2.68–2.73, 3.07–3.24, 3.58–3.64 ppm) were excluded before integration to avoid the presence of potentially confounding factors in multivariate analyses^41,46,47^. The multivariate statistical analysis was performed using both CPMG and NOESY binned spectra.

Different kinds of multivariate statistical techniques were used on the obtained bins using R 3.0.2 in house scripts^41^.

Unsupervised Principal Component Analysis (PCA) was used to obtain a preliminary outlook of the data (visualization in reduced space, cluster detection, screening for outliers). Partial Least Squares (PLS) analysis was employed to perform supervised data reduction and classification between samples from healthy and diseased volunteers. Canonical analysis (CA) was used in combination with PLS to increase supervised data reduction and classification. The accuracy for classification was assessed by means of a Monte Carlo validation scheme: each dataset was randomly divided by 200 times into a training set (90% of the data) which was used to build the model and a test set (10% of the data) which was used to test the integrity of the model. The resulting confusion matrix was reported and its discrimination accuracy, specificity and sensitivity were estimated according to standard definitions. Each classification model was also validated using permutation test (n = 500) and the resulting p-value was reported.

Univariate analysis of the NMR data was performed on Fourier transformed and calibrated CPMG spectra. Metabolites, whose peaks in the spectra were well defined and resolved, were assigned and their levels analyzed. The assignment procedure was made up using an ^1^H-NMR spectra library of pure organic compounds, public databases, e.g. Human Metabolome Database^48^, storing reference ^1^H-NMR spectra of metabolites, spiking ^1^H-NMR experiments and using literature data^49^. The relative concentrations of the various metabolites were calculated by integrating the corresponding signals in the spectra^50^, using the AssureNMR Software (Bruker BioSpin) and a home-made tool for R Software.

The nonparametric Wilcoxon-Mann-Whitney test was used for the determination of the meaningful metabolites. Here, a p-value < 0.05 was considered statistically significant. Considering FDR, the p-value was corrected using the Benjamini-Hochberg formula and reported as pFDR^51^.

SPSS Statistics (IBM, Version 25 for Mac OS X) was used to perform partial correlation between the level of each metabolite and the levels of all the other metabolites checking for the effect of chronological age (at the moment of blood-collection). We considered r-value between 0.4 and 0.7 as moderate correlation and r > 0.7 as strong correlation^52^. Briefly, from the main Menu of the “SPSS Statistics” software, we selected “Analyze” and then “Correlate”; we chose “Partial...” and finally we inserted our data in the main box and inserted “Age at Blood Collection” in the “Controlling for” box. To obtain p-value after FDR correction, we created a file with all the p-values obtained from the previous analysis and, using JMP software (SAS Institute, Version 14), from the main menu we selected “Add-in”, then “False Discovery Rate P-value” command and finally inserted the p-value column in “PValue column”.

To analyze the correlations between metabolite levels and AE scores obtained from Griffiths-III and WPPSI-III tests, we performed a partial correlation checking for the effect of chronological age (at the moment of the cognitive test) using SPSS Statistics software (from the main software Menu we selected “Analyze” and then “Correlate”, then “Partial...” and finally we included our data in the main box and inserted “Age” in the “Controlling for” box).

To investigate the influence of the different IQ scores on the metabolomic profiles, we divided the metabolome results into two groups: those deriving from subjects with DS having taken the Griffiths-III test and those deriving from subjects with DS having taken the WPPSI-III test. We distinguished the subjects according to the IQ scores obtained from the two kinds of cognitive tests for both groups. It is known that the IQ scores have a mean of 100 and an SD of 15 and that a subject with an IQ < −2SD has an intellectual disability. All subjects with DS included in this study have an IQ < −2SD. To perform the multivariate statistical analysis between a significant number of metabolomic profiles for each kind of cognitive test, we decided to create two main groups of data: a group of metabolomic profiles from subjects with an IQ > 40 (between 2 and 4 SD below average) and a second group from subjects with an IQ ≤ 40 (more than 4 SD below average).

## Supplementary information

Supplementary information.

Supplementary information.

## Data Availability

The datasets generated and analyzed during the current study have been made available as Supplementary Dataset S1 (plasma metabolome and cognitive data).
