# Supplementary material for: Plasma metabolome and cognitive skills in Down syndrome
Source: Sci Rep. 2020 Jun 26;10:10491. doi: 10.1038/s41598-020-67195-z (PMC7319960; doi:10.1038/s41598-020-67195-z)
Supplement: Supplementary file 2 — Supplementary information. [file 41598_2020_67195_MOESM2_ESM.pdf]

Supplementary Material

Plasma metabolome and cognitive skills in Down syndrome

Francesca Antonaros[1†], Veronica Ghini[†2], Francesca Pulina[†3], Giuseppe Ramacieri[1], Elena Cicchini[1], Elisa Mannini[1], Anna Martelli[4], Agnese Feliciello[4], Silvia Lanfranchi[3], Sara Onnivello[3], Renzo Vianello[3], Chiara Locatelli[5], Guido Cocchi[4], Maria Chiara Pelleri[1], Lorenza Vitale[1], Pierluigi Strippoli[1], Claudio Luchinat[6], Paola Turano[6*], Allison Piovesan[1*], Maria Caracausi[1]

† These Authors equally contributed to the work

[1]Department of Experimental, Diagnostic and Specialty Medicine, (DIMES), Unit of Histology, Embryology and Applied Biology, University of Bologna, Via Belmeloro 8, 40126 Bologna, BO, Italy.

[2]CERM, Center of Magnetic Resonance, University of Florence, via Luigi Sacconi 6, 50019 Sesto Fiorentino, Florence, Italy. CIRMMP, Consorzio Interuniversitario Risonanze Magnetiche metallo proteine, via Luigi Sacconi 6, 50019 Sesto Fiorentino, Florence, FI, Italy.

[3]Department of Developmental Psychology and Socialisation, University of Padova, Via Venezia 8, 35131, Padova, PD, Italy

[4]Neonatology Unit, St. Orsola-Malpighi Polyclinic, Department of Medical and Surgical Sciences (DIMEC), University of Bologna, Via Massarenti 9, 40138 Bologna, BO, Italy.

[5]Neonatology Unit, St. Orsola-Malpighi Polyclinic, Via Massarenti 9, 40138 Bologna, BO, Italy.

[6]Department of chemistry, University of Florence, via della Lastruccia 3, 50019 Sesto Fiorentino, Florence, FI, Italy.

*Corresponding Authors

E-mail: turano@cerm.unifi.it

E-mail: allison.piovesan2@unibo.it

Supplementary Figure S1. Representative PLS-CA analysis of a random selection of 46 DS and 46 CTRL samples derived from all plasma samples, repeated 500 times: **(A)** CPMG and **(B)** NOESY spectra. **Representative PLS-CA analysis of a random selection of 35 DS and 35 CTRL samples derived from plasma samples of fasting subjects**, repeated 500 times: **(C)** CPMG and **(D)** NOESY spectra. In the score plots each dot represents a different plasma sample. Orange dots: Down syndrome samples; blue dots: healthy controls.

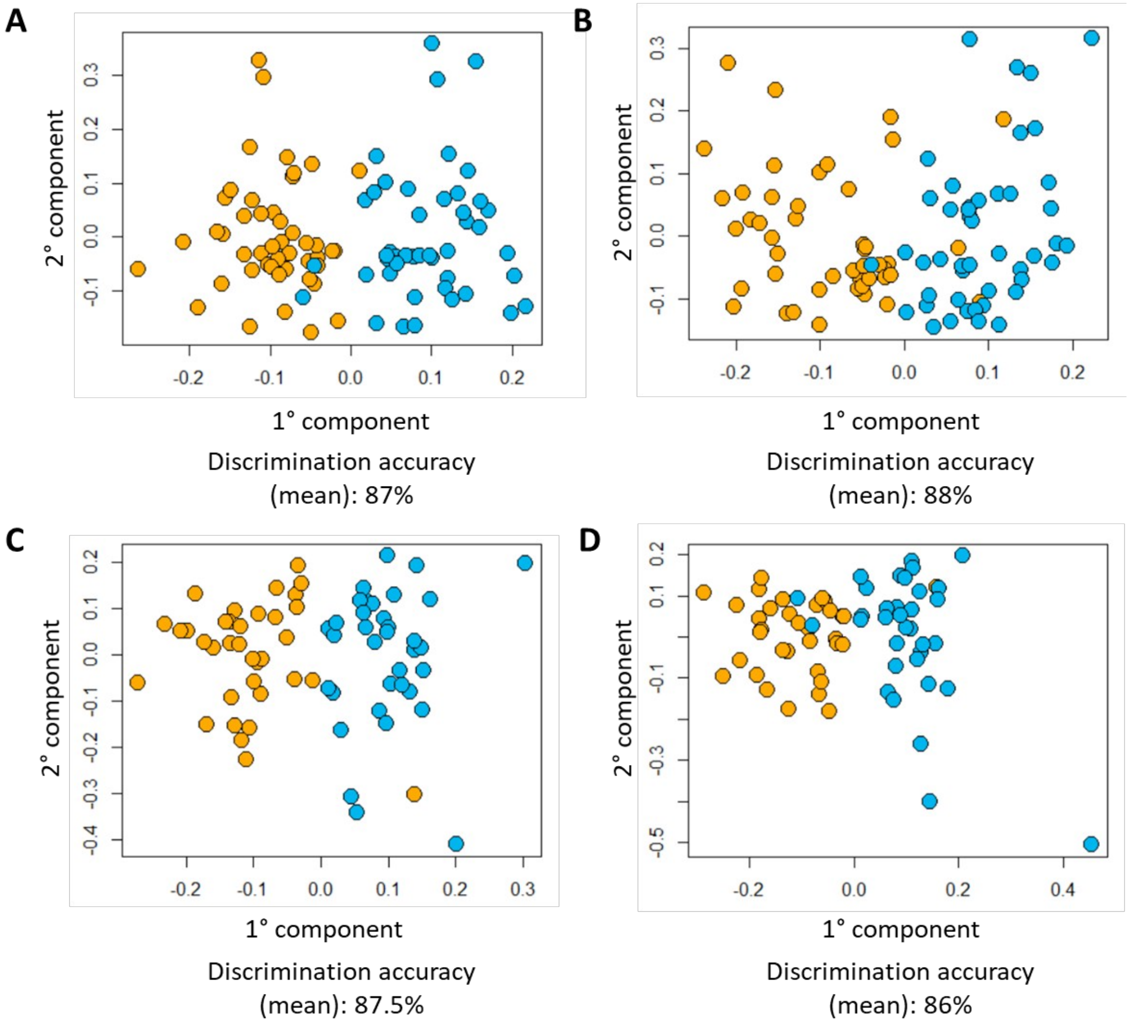

Supplementary Figure S2. Representative PLS-CA analysis of a random selection of 22 DS and 22 CTRL samples derived from plasma samples of female subjects, repeated 500 times: **(A)** CPMG and **(B)** NOESY spectra. **Representative PLS-CA analysis of a random selection of 24 DS and 24 CTRL samples derived from plasma samples of male subjects**, repeated 500 times: **(C)** CPMG and **(D)** NOESY spectra. In the score plots each dot represents a different plasma sample. Orange dots: Down syndrome samples; blue dots: healthy controls.

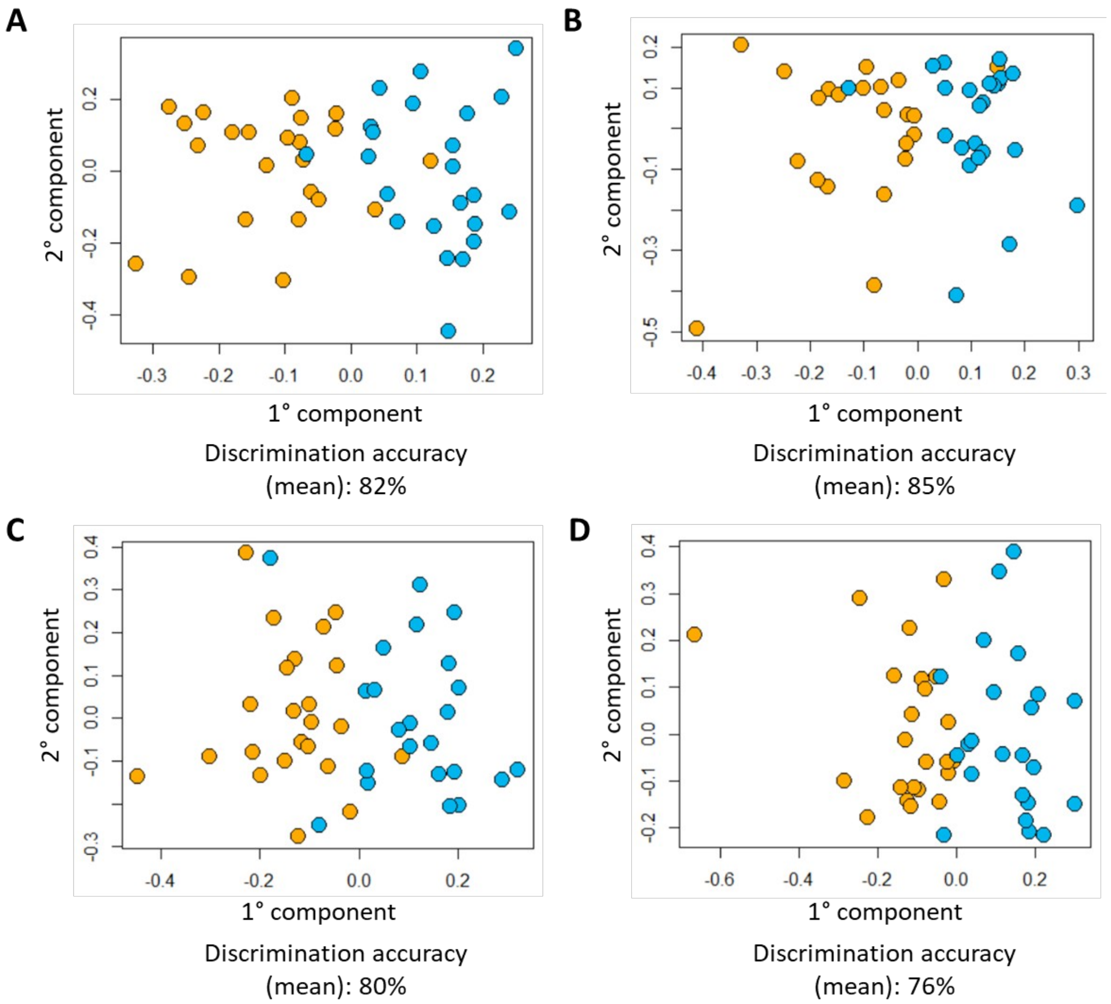

Supplementary Figure S3. PLS-CA analysis to investigate if NMR-detectable part of plasma metabolome of DS patients contains signatures of the different IQ scores obtained by WPPSI tests. Discrimination between samples from DS subjects with an IQ>40 (n=13) and from DS subjects an IQ≤40 (n=26). Score plot, each dot represents a different plasma sample. Orange dots: samples from DS subjects with an IQ>40; blue dots: samples from DS subjects with an IQ≤40. Confusion matrix and discrimination accuracy are also reported.

WPPSI test

	IQ > 40	IQ < 40
IQ > 40	**63**	37
IQ < 40	20	**80**

Discrimination Accuracy=74.5%

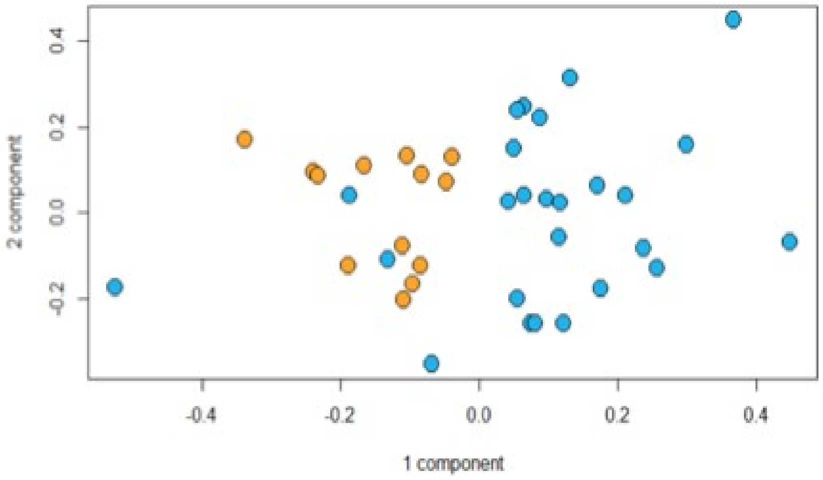

Supplementary Figure S4. PLS-CA analysis to investigate if NMR-detectable part of plasma metabolome of DS patients contains signatures of the different IQ disability ranges obtained by Griffiths-III tests. Discrimination between samples from DS subjects with an IQ>40 (n=17) and from DS subjects an IQ≤40 (n=5). Score plot, each dot represents a different plasma sample. Orange dots: samples from DS subjects with an IQ>40; blue dots: samples from DS subjects with an IQ≤40. Confusion matrix and discrimination accuracy are also reported.

Griffiths test

	IQ > 40	IQ < 40
IQ > 40	**85**	15
IQ < 40	61	**39**

Discrimination Accuracy=74.3%

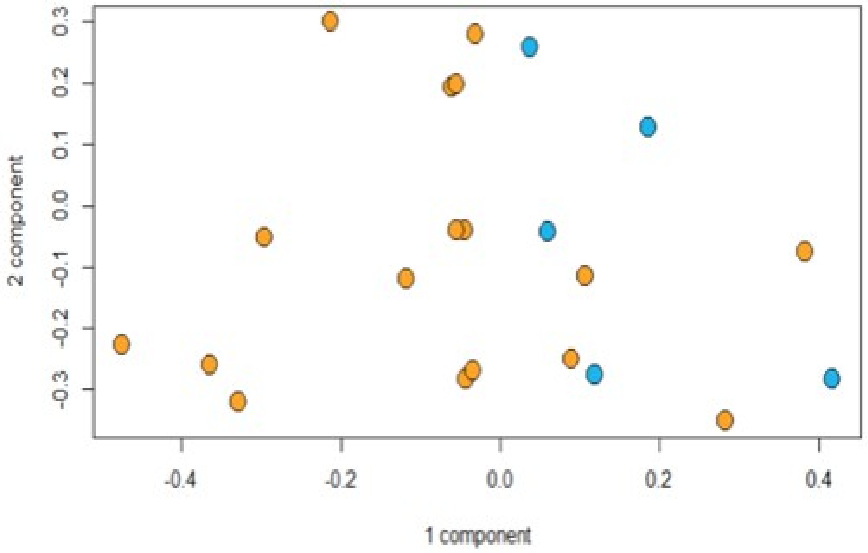

Supplementary Table 1. Multivariate statistical analysis of correlations between metabolites involved in Krebs cycle and other metabolites (DS, n=129; CTRL, n=46). The table contains the statistically significant correlations (in DS and/or CTRL group) between metabolites analyzed with 1H-NMR from plasma samples. We reported p-value of the univariate Wilcoxon test, p-value after FDR correction and r for each metabolite. Correlations with significant p-value after FDR correction (<0.01) are highlighted in red. Pairs with moderate correlation (0.4< r <0.7) are highlighted in green and pairs with strong correlation (r >0.7) are highlighted in blue.

Metabolite 1	Metabolite 2	DS Metabolites			CTRL Metabolites		
		P-value	P-value after FDR correction	r	P-value	P-value after FDR correction	r
pyruvate	leucine	< 0.001	< 0.001	0.3135	0.361	0.523	0.1379
pyruvate	valine	0.001	0.003	0.2831	< 0.001	< 0.001	0.5755
pyruvate	alanine	0.006	0.014	0.2388	< 0.001	< 0.001	0.6332
pyruvate	acetone	< 0.001	< 0.001	0.3086	0.167	0.291	0.2074
pyruvate	glutamine	0.021	0.041	0.2030	0.001	0.005	0.4880
pyruvate	citrate	< 0.001	< 0.001	0.3257	0.006	0.022	0.3978
pyruvate	glycine	0.001	0.003	0.2876	< 0.001	< 0.001	0.6366
pyruvate	creatine	< 0.001	< 0.001	0.3387	0.042	0.103	-0.3009
pyruvate	creatinine	0.016	0.032	0.2122	< 0.001	< 0.001	0.4951
pyruvate	lactate	< 0.001	< 0.001	0.5644	< 0.001	< 0.001	0.6894
pyruvate	glucose	0.651	0.728	0.0402	< 0.001	< 0.001	0.6335
pyruvate	mannose	0.904	0.932	0.0108	0.005	0.019	0.4031
pyruvate	tyrosine	0.362	0.444	-0.0808	0.002	0.009	0.4370
pyruvate	histidine	0.004	0.010	0.2537	< 0.001	< 0.001	0.6441
pyruvate	fumarate	0.010	0.021	0.2273	0.058	0.133	0.2817
pyruvate	threonine	0.866	0.901	0.0150	0.007	0.025	0.3952
pyruvate	lysine	< 0.001	< 0.001	0.3435	0.256	0.401	-0.1710
pyruvate	methionine	0.016	0.032	0.2108	< 0.001	< 0.001	0.5775
pyruvate	unk1	0.021	0.041	0.2035	0.971	0.991	0.0056
pyruvate	unk2	< 0.001	< 0.001	0.5198	0.064	0.142	0.2756
pyruvate	3-hydroxybutyrate	0.007	0.016	0.2347	0.170	0.295	-0.2060
pyruvate	succinate	< 0.001	< 0.001	0.3795	< 0.001	< 0.001	-0.5426
pyruvate	2-hydroxybutyrate	< 0.001	< 0.001	0.3687	0.572	0.713	0.0854
pyruvate	proline	< 0.001	< 0.001	0.3281	< 0.001	< 0.001	-0.5362
citrate	leucine	< 0.001	< 0.001	0.3179	0.391	0.553	0.1296
citrate	valine	0.006	0.014	0.2405	0.001	0.005	0.4788
citrate	alanine	0.029	0.054	0.1923	0.016	0.049	0.3527
citrate	acetate	0.006	0.014	0.2409	0.001	0.005	0.4919
citrate	pyruvate	< 0.001	< 0.001	0.3257	0.006	0.022	0.3978
citrate	acetone	< 0.001	< 0.001	0.3131	0.033	0.085	0.3149
citrate	glutamine	< 0.001	< 0.001	0.4807	0.005	0.019	0.4052
citrate	glycine	< 0.001	< 0.001	0.3514	0.005	0.019	0.4092
citrate	creatine	0.001	0.003	0.2774	0.484	0.634	-0.1059
citrate	creatinine	< 0.001	< 0.001	0.3410	0.072	0.156	0.2673
citrate	lactate	0.539	0.625	0.0546	< 0.001	< 0.001	0.6602
citrate	glucose	< 0.001	< 0.001	0.3420	< 0.001	< 0.001	0.6215
citrate	histidine	< 0.001	< 0.001	0.4358	0.002	0.009	0.4409
citrate	formate	0.029	0.054	0.1918	0.002	0.009	0.4429
citrate	fumarate	0.014	0.029	0.2168	0.012	0.039	0.3689
citrate	lysine	< 0.001	< 0.001	0.3607	0.223	0.364	-0.1833
citrate	acetatoacetate	< 0.001	< 0.001	0.3206	0.050	0.119	0.2905
citrate	methionine	0.163	0.230	0.1234	< 0.001	< 0.001	0.5093
citrate	unk1	< 0.001	< 0.001	0.3662	0.002	0.009	0.4364
citrate	unk2	0.759	0.813	-0.0272	0.031	0.083	0.3188
citrate	unk3	0.002	0.005	0.2649	0.553	0.699	-0.0898
citrate	3-hydroxybutyrate	< 0.001	< 0.001	0.3366	0.770	0.859	0.0443
citrate	succinate	< 0.001	< 0.001	0.3390	0.009	0.031	-0.3785
citrate	2-hydroxybutyrate	< 0.001	< 0.001	0.4666	0.001	0.005	0.4586
citrate	proline	0.003	0.008	0.2572	0.019	0.056	-0.3447
succinate	leucine	0.007	0.016	0.2362	0.055	0.127	0.2850
succinate	isoleucine	0.234	0.303	0.1056	< 0.001	< 0.001	0.5689
succinate	valine	0.001	0.003	0.2770	< 0.001	< 0.001	-0.5128
succinate	alanine	0.040	0.071	0.1814	< 0.001	< 0.001	-0.5245
succinate	acetate	0.005	0.012	0.2456	0.041	0.102	-0.3023
succinate	pyruvate	< 0.001	< 0.001	0.3795	< 0.001	< 0.001	-0.5426
succinate	glutamine	< 0.001	< 0.001	0.3457	0.766	0.856	-0.0451
succinate	citrate	< 0.001	< 0.001	0.3390	0.009	0.031	-0.3785
succinate	glycine	< 0.001	< 0.001	0.3963	0.005	0.019	-0.4096
succinate	creatine	0.002	0.005	0.2765	< 0.001	< 0.001	0.7243
succinate	creatinine	0.017	0.034	0.2097	0.096	0.193	-0.2486
succinate	lactate	< 0.001	< 0.001	0.4360	0.002	0.009	-0.4375
succinate	glucose	0.001	0.003	0.2884	< 0.001	< 0.001	-0.5852

succinate	mannose	0.077	0.122	0.1562	0.015	0.048	-0.3574
succinate	histidine	0.006	0.014	0.2410	0.019	0.056	-0.3453
succinate	formate	0.009	0.019	0.2301	0.095	0.192	-0.2494
succinate	fumarate	0.007	0.016	0.2380	0.882	0.940	-0.0224
succinate	lysine	0.002	0.005	0.2703	< 0.001	< 0.001	0.8437
succinate	methionine	0.094	0.145	0.1479	< 0.001	< 0.001	-0.5143
succinate	unk1	0.001	0.003	0.2822	0.209	0.350	0.1886
succinate	unk2	< 0.001	< 0.001	0.3916	0.663	0.788	0.0659
succinate	unk3	< 0.001	< 0.001	0.3527	0.007	0.025	0.3927
succinate	3-hydroxybutyrate	0.011	0.023	0.2227	< 0.001	< 0.001	0.6073
succinate	2-hydroxybutyrate	0.001	0.003	0.2775	0.852	0.921	0.0283
succinate	proline	0.001	0.003	0.2894	< 0.001	< 0.001	0.9766
fumarate	pyruvate	0.010	0.021	0.2273	0.058	0.133	0.2817
fumarate	glutamine	0.063	0.105	0.1643	0.004	0.016	0.4192
fumarate	citrate	0.014	0.029	0.2168	0.012	0.039	0.3689
fumarate	glycine	0.001	0.003	0.2776	0.217	0.357	0.1856
fumarate	lactate	0.006	0.014	0.2421	0.016	0.049	0.3545
fumarate	mannose	0.002	0.005	0.2751	0.546	0.696	0.0914
fumarate	threonine	0.018	0.036	0.2086	0.663	0.788	0.0659
fumarate	unk2	0.009	0.019	0.2282	0.108	0.214	0.2401
fumarate	succinate	0.007	0.016	0.2380	0.882	0.940	-0.0224

Supplementary Table 2. Multivariate statistical analysis of correlations between formate and other metabolites (DS, n=129; CTRL, n=46). The table contains the statistically significant correlations (in DS and/or CTRL group) between metabolites analyzed with 1H-NMR from plasma samples. We reported p-value of the univariate Wilcoxon test, p-value after FDR correction and r for each metabolite. Correlations with significant p-value after FDR correction (<0.01) are highlighted in red. Pairs with moderate correlation (0.4< r <0.7) are highlighted in green and pairs with strong correlation (r >0.7) are highlighted in blue.

Metabolite 1	Metabolite 2	DS Metabolites			CTRL Metabolites		
		P-value	P-value after FDR correction	r	P-value	P-value after FDR correction	r
formate	acetate	< 0.001	< 0.001	0.6349	< 0.001	< 0.001	0.7798
formate	acetone	0.183	0.248	0.1180	0.001	0.005	0.4690
formate	citrate	0.029	0.054	0.1918	0.002	0.009	0.4429
formate	glycine	0.006	0.014	0.2394	0.064	0.142	0.2753
formate	lysine	0.018	0.036	0.2078	0.403	0.556	-0.1262
formate	acetatoacetate	0.069	0.113	0.1607	0.001	0.005	0.4665
formate	methionine	0.608	0.688	0.0456	0.005	0.019	0.4092
formate	unk1	0.005	0.012	0.2436	0.016	0.049	0.3532
formate	unk3	< 0.001	< 0.001	0.4300	0.037	0.094	0.3086
formate	succinate	0.009	0.019	0.2301	0.095	0.192	-0.2494
formate	2-hydroxybutyrate	0.028	0.052	0.1933	< 0.001	< 0.001	0.4968

Supplementary Table 3. Multivariate statistical analysis of correlations between ketone bodies and other metabolites (DS, n=129; CTRL, n=46). The table contains the statistically significant correlations (in DS and/or CTRL group) between metabolites analyzed with 1H-NMR from plasma samples. We reported p-value of the univariate Wilcoxon test, p-value after FDR correction and r for each metabolite. Correlations with significant p-value after FDR correction (<0.01) are highlighted in red. Pairs with moderate correlation (0.4< r <0.7) are highlighted in green and pairs with strong correlation (r >0.7) are highlighted in blue.

Metabolite 1	Metabolite 2	DS Metabolites			CTRL Metabolites		
		P-value	P-value after FDR correction	r	P-value	P-value after FDR correction	r
2-hydroxybutyrate	leucine	< 0.001	< 0.001	0.3882	0.064	0.142	0.2751
2-hydroxybutyrate	valine	< 0.001	< 0.001	0.3193	0.251	0.394	0.1728
2-hydroxybutyrate	alanine	0.670	0.738	-0.0378	0.007	0.025	-0.3894
2-hydroxybutyrate	acetate	0.020	0.039	0.2047	0.004	0.016	0.4209
2-hydroxybutyrate	pyruvate	< 0.001	< 0.001	0.3687	0.572	0.713	0.0854
2-hydroxybutyrate	acetone	< 0.001	< 0.001	0.4966	< 0.001	< 0.001	0.7761
2-hydroxybutyrate	glutamine	< 0.001	< 0.001	0.3273	0.963	0.986	-0.0070
2-hydroxybutyrate	citrate	< 0.001	< 0.001	0.4666	0.001	0.005	0.4586
2-hydroxybutyrate	glycine	0.002	0.005	0.2674	0.897	0.950	0.0197
2-hydroxybutyrate	creatine	< 0.001	< 0.001	0.5004	0.045	0.108	0.2975
2-hydroxybutyrate	creatinine	0.001	0.003	0.3008	0.909	0.958	-0.0174
2-hydroxybutyrate	glucose	< 0.001	< 0.001	0.3610	0.549	0.697	0.0908
2-hydroxybutyrate	mannose	0.002	0.005	0.2742	0.522	0.676	0.0968
2-hydroxybutyrate	formate	0.028	0.052	0.1933	< 0.001	< 0.001	0.4968
2-hydroxybutyrate	lysine	< 0.001	< 0.001	0.5244	0.346	0.507	0.1422
2-hydroxybutyrate	acetatoacetate	< 0.001	< 0.001	0.6031	< 0.001	< 0.001	0.7921
2-hydroxybutyrate	methionine	< 0.001	< 0.001	0.4144	0.292	0.448	0.1589
2-hydroxybutyrate	unk1	< 0.001	< 0.001	0.4077	< 0.001	< 0.001	0.5206
2-hydroxybutyrate	unk3	< 0.001	< 0.001	0.3528	0.055	0.127	0.2845
2-hydroxybutyrate	3-hydroxybutyrate	< 0.001	< 0.001	0.5532	< 0.001	< 0.001	0.6708
2-hydroxybutyrate	succinate	0.001	0.003	0.2775	0.852	0.921	0.0283
2-hydroxybutyrate	proline	0.001	0.003	0.2844	0.371	0.532	0.1351
3-hydroxybutyrate	leucine	0.021	0.041	0.2027	0.010	0.034	0.3769
3-hydroxybutyrate	isoleucine	0.997	0.997	0.0003	0.002	0.009	0.4504
3-hydroxybutyrate	alanine	0.022	0.042	-0.2019	< 0.001	< 0.001	-0.5827
3-hydroxybutyrate	acetate	0.009	0.019	0.2291	0.816	0.887	0.0353
3-hydroxybutyrate	pyruvate	0.007	0.016	0.2347	0.170	0.295	-0.2060
3-hydroxybutyrate	acetone	< 0.001	< 0.001	0.8244	< 0.001	< 0.001	0.6605
3-hydroxybutyrate	citrate	< 0.001	< 0.001	0.3366	0.770	0.859	0.0443
3-hydroxybutyrate	creatine	0.003	0.008	0.2589	< 0.001	< 0.001	0.5849
3-hydroxybutyrate	creatinine	0.015	0.031	0.2137	0.338	0.499	-0.1445
3-hydroxybutyrate	tyrosine	0.203	0.267	-0.1129	0.001	0.005	-0.4644
3-hydroxybutyrate	lysine	0.003	0.008	0.2628	< 0.001	< 0.001	0.5895
3-hydroxybutyrate	acetatoacetate	< 0.001	< 0.001	0.8765	< 0.001	< 0.001	0.7065
3-hydroxybutyrate	unk1	0.002	0.005	0.2715	0.001	0.005	0.4711
3-hydroxybutyrate	unk3	0.071	0.115	0.1595	0.006	0.022	0.3957
3-hydroxybutyrate	succinate	0.011	0.023	0.2227	< 0.001	< 0.001	0.6073
3-hydroxybutyrate	2-hydroxybutyrate	< 0.001	< 0.001	0.5532	< 0.001	< 0.001	0.6708
3-hydroxybutyrate	proline	< 0.001	< 0.001	0.5540	< 0.001	< 0.001	0.7047
acetate	leucine	< 0.001	< 0.001	0.3023	0.885	0.940	-0.0220
acetate	valine	< 0.001	< 0.001	0.3203	0.192	0.329	0.1958
acetate	glutamine	< 0.001	< 0.001	0.3691	0.396	0.554	0.1283
acetate	citrate	0.006	0.014	0.2409	0.001	0.005	0.4919
acetate	creatinine	0.003	0.008	0.2630	0.914	0.958	-0.0164
acetate	glucose	< 0.001	< 0.001	0.3288	0.041	0.102	0.3029
acetate	tyrosine	0.012	0.025	0.2200	0.805	0.880	0.0374
acetate	histidine	0.004	0.010	0.2520	0.425	0.581	0.121
acetate	formate	< 0.001	< 0.001	0.6349	< 0.001	< 0.001	0.7798
acetate	threonine	0.013	0.027	0.2181	0.211	0.350	-0.1880
acetate	lysine	< 0.001	< 0.001	0.3228	0.081	0.171	-0.260
acetate	acetatoacetate	0.009	0.019	0.2308	0.017	0.051	0.3511
acetate	methionine	0.656	0.730	0.0396	0.010	0.034	0.376
acetate	unk1	0.004	0.010	0.2551	0.016	0.049	0.3531
acetate	unk3	< 0.001	< 0.001	0.3914	0.678	0.798	-0.0629
acetate	3-hydroxybutyrate	0.009	0.019	0.2291	0.816	0.887	0.0353
acetate	succinate	0.005	0.012	0.2456	0.041	0.102	-0.3023
acetate	2-hydroxybutyrate	0.020	0.039	0.2047	0.004	0.016	0.4209
acetate	proline	< 0.001	< 0.001	0.3043	0.093	0.189	-0.2503
acetone	alanine	< 0.001	< 0.001	-0.3213	0.086	0.179	-0.2561
acetone	pyruvate	< 0.001	< 0.001	0.3086	0.167	0.291	0.2074
acetone	citrate	< 0.001	< 0.001	0.3131	0.033	0.085	0.3149
acetone	tyrosine	0.023	0.044	-0.1997	0.114	0.224	-0.2364

acetone	formate	0.183	0.248	0.1180	0.001	0.005	0.4690
acetone	acetatoacetate	< 0.001	< 0.001	0.8999	< 0.001	< 0.001	0.9664
acetone	unk1	0.003	0.008	0.2616	0.009	0.031	0.3828
acetone	3-hydroxybutyrate	< 0.001	< 0.001	0.8244	< 0.001	< 0.001	0.6605
acetone	2-hydroxybutyrate	< 0.001	< 0.001	0.4966	< 0.001	< 0.001	0.7761
acetone	proline	< 0.001	< 0.001	0.4827	0.933	0.966	-0.0127
acetatoacetate	alanine	< 0.001	< 0.001	-0.3134	0.027	0.075	-0.3260
acetatoacetate	acetate	0.009	0.019	0.2308	0.017	0.051	0.3511
acetatoacetate	acetone	< 0.001	< 0.001	0.8999	< 0.001	< 0.001	0.9664
acetatoacetate	citrate	< 0.001	< 0.001	0.3206	0.050	0.119	0.2905
acetatoacetate	phenylalanine	0.023	0.044	-0.1996	0.033	0.085	-0.3141
acetatoacetate	methionine	0.012	0.025	0.2217	0.193	0.329	0.1955
acetatoacetate	unk1	0.002	0.005	0.2698	0.009	0.031	0.3790
acetatoacetate	3-hydroxybutyrate	< 0.001	< 0.001	0.8765	< 0.001	< 0.001	0.7065
acetatoacetate	2-hydroxybutyrate	< 0.001	< 0.001	0.6031	< 0.001	< 0.001	0.7921
acetatoacetate	proline	< 0.001	< 0.001	0.5202	0.680	0.798	0.0624

Supplementary Table 4. Multivariate statistical analysis of correlations between lactate, glucose, mannose and other metabolites (DS, n=129; CTRL, n=46). The table contains the statistically significant correlations (in DS and/or CTRL group) between metabolites analyzed with 1H-NMR from plasma samples. We reported p-value of the univariate Wilcoxon test, p-value after FDR correction and r for each metabolite. Correlations with significant p-value after FDR correction (<0.01) are highlighted in red. Pairs with moderate correlation (0.4< r <0.7) are highlighted in green and pairs with strong correlation (r >0.7) are highlighted in blue.

Metabolite 1	Metabolite 2	DS Metabolites			CTRL Metabolites		
		P-value	P-value after FDR correction	r	P-value	P-value after FDR correction	r
lactate	valine	0.240	0.309	0.1041	0.001	0.005	0.4633
lactate	alanine	0.001	0.003	0.2794	< 0.001	< 0.001	0.5742
lactate	pyruvate	< 0.001	< 0.001	0.5644	< 0.001	< 0.001	0.6894
lactate	glutamine	0.598	0.678	-0.0469	0.001	0.005	0.4553
lactate	citrate	0.539	0.625	0.0546	< 0.001	< 0.001	0.6602
lactate	glycine	0.437	0.524	0.0690	< 0.001	< 0.001	0.5434
lactate	glucose	0.491	0.578	0.0611	< 0.001	< 0.001	0.5955
lactate	tyrosine	0.237	0.306	-0.1048	0.004	0.016	0.4178
lactate	histidine	0.530	0.618	0.0558	< 0.001	< 0.001	0.5307
lactate	fumarate	0.006	0.014	0.2421	0.016	0.049	0.3545
lactate	methionine	0.848	0.892	0.0171	< 0.001	< 0.001	0.5570
lactate	unk2	< 0.001	< 0.001	0.9157	< 0.001	< 0.001	0.6706
lactate	succinate	< 0.001	< 0.001	0.4360	0.002	0.009	-0.4375
lactate	proline	0.001	0.003	0.2871	0.007	0.025	-0.3939
glucose	leucine	< 0.001	< 0.001	0.4602	0.861	0.927	0.0266
glucose	isoleucine	0.009	0.019	0.2278	0.065	0.143	-0.2748
glucose	valine	< 0.001	< 0.001	0.4779	< 0.001	< 0.001	0.5285
glucose	alanine	< 0.001	< 0.001	0.3946	< 0.001	< 0.001	0.6576
glucose	acetate	< 0.001	< 0.001	0.3288	0.041	0.102	0.3029
glucose	pyruvate	0.651	0.728	0.0402	< 0.001	< 0.001	0.6335
glucose	glutamine	< 0.001	< 0.001	0.6416	< 0.001	< 0.001	0.6545
glucose	citrate	< 0.001	< 0.001	0.3420	< 0.001	< 0.001	0.6215
glucose	glycine	< 0.001	< 0.001	0.5412	< 0.001	< 0.001	0.7217
glucose	creatine	< 0.001	< 0.001	0.3891	0.020	0.058	-0.3427
glucose	creatinine	< 0.001	< 0.001	0.3862	< 0.001	< 0.001	0.5158
glucose	lactate	0.491	0.578	0.0611	< 0.001	< 0.001	0.5955
glucose	mannose	< 0.001	< 0.001	0.4064	0.003	0.013	0.4342
glucose	tyrosine	< 0.001	< 0.001	0.3153	0.001	0.005	0.4614
glucose	histidine	< 0.001	< 0.001	0.3812	< 0.001	< 0.001	0.6141
glucose	phenylalanine	0.004	0.010	0.2539	0.991	0.996	0.0017
glucose	lysine	< 0.001	< 0.001	0.6239	0.069	0.150	-0.2706
glucose	methionine	< 0.001	< 0.001	0.3232	< 0.001	< 0.001	0.6628
glucose	unk1	< 0.001	< 0.001	0.3245	0.672	0.795	-0.0642
glucose	unk3	< 0.001	< 0.001	0.3429	0.576	0.715	0.0846
glucose	succinate	0.001	0.003	0.2884	< 0.001	< 0.001	-0.5852
glucose	2-hydroxybutyrate	< 0.001	< 0.001	0.3610	0.549	0.697	0.0908
glucose	proline	< 0.001	< 0.001	0.3076	< 0.001	< 0.001	-0.6027
mannose	leucine	< 0.001	< 0.001	0.3738	0.636	0.766	-0.0716
mannose	isoleucine	0.001	0.003	0.2774	0.031	0.083	-0.3193
mannose	valine	< 0.001	< 0.001	0.4010	0.160	0.284	0.2109
mannose	pyruvate	0.904	0.932	0.0108	0.005	0.019	0.4031
mannose	glutamine	0.004	0.010	0.2526	0.084	0.177	0.2571
mannose	creatine	0.012	0.025	0.2219	0.243	0.387	-0.1755
mannose	creatinine	0.025	0.047	0.1977	0.119	0.232	0.2333
mannose	glucose	< 0.001	< 0.001	0.4064	0.003	0.013	0.4342
mannose	tyrosine	0.005	0.012	0.2482	0.008	0.028	0.3888
mannose	fumarate	0.002	0.005	0.2751	0.546	0.696	0.0914
mannose	threonine	0.006	0.014	0.2422	0.085	0.178	0.2568
mannose	lysine	< 0.001	< 0.001	0.4136	0.333	0.493	-0.1459
mannose	methionine	0.014	0.029	0.2158	0.075	0.161	0.2647
mannose	unk1	0.003	0.008	0.2607	0.450	0.608	-0.1140
mannose	unk3	0.001	0.003	0.2951	0.320	0.483	0.1499
mannose	succinate	0.077	0.122	0.1562	0.015	0.048	-0.3574
mannose	2-hydroxybutyrate	0.002	0.005	0.2742	0.522	0.676	0.0968
mannose	proline	0.253	0.323	0.1015	0.012	0.039	-0.3667

Supplementary Table 5. Multivariate statistical analysis of correlation between branched-chain amino acids (BCAA) and other metabolites (DS, n=129; CTRL, n=46). The table contains the statistically significant correlations (in DS and/or CTRL group) between metabolites analyzed with 1H-NMR from plasma samples. We reported p-value of the univariate Wilcoxon test, p-value after FDR correction and r for each metabolite. Correlations with significant p-value after FDR correction (<0.01) are highlighted in red. Pairs with moderate correlation (0.4< r <0.7) are highlighted in green and pairs with strong correlation (r >0.7) are highlighted in blue.

Metabolite 1	Metabolite 2	DS Metabolites			CTRL Metabolites		
		P-value	P-value after FDR correction	r	P-value	P-value after FDR correction	r
leucine	isoleucine	< 0.001	< 0.001	0.8265	< 0.001	< 0.001	0.8496
leucine	valine	< 0.001	< 0.001	0.9352	< 0.001	< 0.001	0.6086
leucine	alanine	0.001	0.003	0.2855	0.883	0.940	0.0222
leucine	acetate	< 0.001	< 0.001	0.3023	0.885	0.940	-0.0220
leucine	pyruvate	< 0.001	< 0.001	0.3135	0.361	0.523	0.1379
leucine	glutamine	< 0.001	< 0.001	0.5966	0.005	0.019	0.4079
leucine	citrate	< 0.001	< 0.001	0.3179	0.391	0.553	0.1296
leucine	glycine	0.001	0.003	0.3017	0.323	0.484	0.1490
leucine	creatine	< 0.001	< 0.001	0.4035	0.024	0.068	0.3332
leucine	creatinine	< 0.001	< 0.001	0.5598	0.001	0.005	0.4872
leucine	glucose	< 0.001	< 0.001	0.4602	0.861	0.927	0.0266
leucine	mannose	< 0.001	< 0.001	0.3738	0.636	0.766	-0.0716
leucine	tyrosine	0.001	0.003	0.2819	0.029	0.079	0.3225
leucine	histidine	< 0.001	< 0.001	0.4725	0.048	0.114	0.2930
leucine	phenylalanine	< 0.001	< 0.001	0.4165	0.126	0.240	0.2286
leucine	lysine	< 0.001	< 0.001	0.8479	< 0.001	< 0.001	0.6494
leucine	methionine	< 0.001	< 0.001	0.3528	0.127	0.240	0.2280
leucine	unk1	< 0.001	< 0.001	0.5247	0.012	0.039	0.3657
leucine	unk3	< 0.001	< 0.001	0.3116	0.010	0.034	0.3778
leucine	3-hydroxybutyrate	0.021	0.041	0.2027	0.010	0.034	0.3769
leucine	succinate	0.007	0.016	0.2362	0.055	0.127	0.2850
leucine	2-hydroxybutyrate	< 0.001	< 0.001	0.3882	0.064	0.142	0.2751
leucine	proline	< 0.001	< 0.001	0.3078	0.032	0.085	0.3170
isoleucine	leucine	< 0.001	< 0.001	0.8265	< 0.001	< 0.001	0.8496
isoleucine	valine	< 0.001	< 0.001	0.7966	0.048	0.114	0.2931
isoleucine	alanine	0.002	0.005	0.2683	0.403	0.556	-0.1264
isoleucine	glutamine	< 0.001	< 0.001	0.4218	0.116	0.227	0.2352
isoleucine	creatine	0.024	0.046	0.1983	0.003	0.013	0.4351
isoleucine	creatinine	< 0.001	< 0.001	0.4235	0.064	0.142	0.2756
isoleucine	glucose	0.009	0.019	0.2278	0.065	0.143	-0.2748
isoleucine	mannose	0.001	0.003	0.2774	0.031	0.083	-0.3193
isoleucine	tyrosine	0.002	0.005	0.2718	0.461	0.616	0.1114
isoleucine	histidine	< 0.001	< 0.001	0.4102	0.458	0.614	0.1121
isoleucine	phenylalanine	< 0.001	< 0.001	0.4122	0.033	0.085	0.3158
isoleucine	threonine	0.011	0.023	0.2223	0.470	0.624	-0.1091
isoleucine	lysine	< 0.001	< 0.001	0.6307	< 0.001	< 0.001	0.7636
isoleucine	unk1	< 0.001	< 0.001	0.3612	0.057	0.131	0.2831
isoleucine	unk3	0.367	0.449	0.0802	0.011	0.037	0.3720
isoleucine	3-hydroxybutyrate	0.997	0.997	0.0003	0.002	0.009	0.4504
isoleucine	succinate	0.234	0.303	0.1056	< 0.001	< 0.001	0.5689
isoleucine	proline	0.033	0.060	0.1878	< 0.001	< 0.001	0.5774
valine	leucine	< 0.001	< 0.001	0.9352	< 0.001	< 0.001	0.6086
valine	isoleucine	< 0.001	< 0.001	0.7966	0.048	0.114	0.2931
valine	alanine	< 0.001	< 0.001	0.3205	0.001	0.005	0.4785
valine	acetate	< 0.001	< 0.001	0.3203	0.192	0.329	0.1958
valine	pyruvate	0.001	0.003	0.2831	< 0.001	< 0.001	0.5755
valine	glutamine	< 0.001	< 0.001	0.5358	0.004	0.016	0.4156
valine	citrate	0.006	0.014	0.2405	0.001	0.005	0.4788
valine	glycine	0.003	0.008	0.2602	< 0.001	< 0.001	0.5527
valine	creatine	< 0.001	< 0.001	0.3831	0.110	0.217	-0.2385
valine	creatinine	< 0.001	< 0.001	0.5087	< 0.001	< 0.001	0.5841
valine	lactate	0.240	0.309	0.1041	0.001	0.005	0.4633
valine	glucose	< 0.001	< 0.001	0.4779	< 0.001	< 0.001	0.5285
valine	mannose	< 0.001	< 0.001	0.4010	0.160	0.284	0.2109
valine	tyrosine	0.001	0.003	0.2821	< 0.001	< 0.001	0.5451
valine	histidine	< 0.001	< 0.001	0.4340	< 0.001	< 0.001	0.6037
valine	phenylalanine	< 0.001	< 0.001	0.4315	0.729	0.837	-0.0525
valine	threonine	0.025	0.047	0.1977	0.086	0.179	0.2561
valine	lysine	< 0.001	< 0.001	0.8100	0.595	0.728	-0.0805
valine	methionine	< 0.001	< 0.001	0.3413	< 0.001	< 0.001	0.6102
valine	unk1	< 0.001	< 0.001	0.5668	0.127	0.240	0.2286
valine	unk3	0.003	0.008	0.2588	0.806	0.880	0.0372

valine	succinate	0.001	0.003	0.2770	< 0.001	< 0.001	-0.5128
valine	2-hydroxybutyrate	< 0.001	< 0.001	0.3193	0.251	0.394	0.1728
valine	proline	< 0.001	0.003	0.2879	0.001	0.005	-0.4883

Supplementary Table 6. Multivariate statistical analysis of correlation between creatine-creatinine and other metabolites (DS, n=129; CTRL, n=46). The table contains the statistically significant correlations (in DS and/or CTRL group) between metabolites analyzed with 1H-NMR from plasma samples. We reported p-value of the univariate Wilcoxon test, p-value after FDR correction and r for each metabolite. Correlations with significant p-value after FDR correction (<0.01) are highlighted in red. Pairs with moderate correlation (0.4< r <0.7) are highlighted in green and pairs with strong correlation (r >0.7) are highlighted in blue.

Metabolite 1	Metabolite 2	DS Metabolites			CTRL Metabolites		
		P-value	P-value after FDR correction	r	P-value	P-value after FDR correction	r
creatine	leucine	< 0.001	< 0.001	0.4035	0.024	0.068	0.3332
creatine	isoleucine	0.024	0.046	0.1983	0.003	0.013	0.4351
creatine	valine	< 0.001	< 0.001	0.3831	0.110	0.217	-0.2385
creatine	alanine	0.300	0.377	0.0919	< 0.001	< 0.001	-0.5290
creatine	pyruvate	< 0.001	< 0.001	0.3387	0.042	0.103	-0.3009
creatine	glutamine	< 0.001	< 0.001	0.3426	0.636	0.766	-0.0716
creatine	citrate	0.001	0.003	0.2774	0.484	0.634	-0.1059
creatine	glycine	< 0.001	< 0.001	0.4132	0.033	0.085	-0.3149
creatine	creatinine	0.015	0.031	0.2141	0.053	0.124	-0.2876
creatine	glucose	< 0.001	< 0.001	0.3891	0.020	0.058	-0.3427
creatine	mannose	0.012	0.025	0.2219	0.243	0.387	-0.1755
creatine	histidine	< 0.001	< 0.001	0.3263	0.091	0.186	-0.2522
creatine	lysine	< 0.001	< 0.001	0.5422	< 0.001	< 0.001	0.7107
creatine	methionine	< 0.001	< 0.001	0.4404	0.054	0.126	-0.2861
creatine	unk1	0.009	0.019	0.2296	0.160	0.284	0.2104
creatine	3-hydroxybutyrate	0.003	0.008	0.2589	< 0.001	< 0.001	0.5849
creatine	succinate	0.002	0.005	0.2765	< 0.001	< 0.001	0.7243
creatine	2-hydroxybutyrate	< 0.001	< 0.001	0.5004	0.045	0.108	0.2975
creatine	proline	0.064	0.106	0.1638	< 0.001	< 0.001	0.7311
creatinine	leucine	< 0.001	< 0.001	0.5598	0.001	0.005	0.4872
creatinine	isoleucine	< 0.001	< 0.001	0.4235	0.064	0.142	0.2756
creatinine	valine	< 0.001	< 0.001	0.5087	< 0.001	< 0.001	0.5841
creatinine	alanine	0.062	0.104	0.1646	0.004	0.016	0.4198
creatinine	acetate	0.003	0.008	0.2630	0.914	0.958	-0.0164
creatinine	pyruvate	0.016	0.032	0.2122	< 0.001	< 0.001	0.4951
creatinine	glutamine	< 0.001	< 0.001	0.5017	< 0.001	< 0.001	0.6029
creatinine	citrate	< 0.001	< 0.001	0.3410	0.072	0.156	0.2673
creatinine	glycine	0.004	0.010	0.2549	< 0.001	< 0.001	0.5763
creatinine	creatine	0.015	0.031	0.2141	0.053	0.124	-0.2876
creatinine	lactate	0.616	0.695	0.0446	0.029	0.079	0.3227
creatinine	glucose	< 0.001	< 0.001	0.3862	< 0.001	< 0.001	0.5158
creatinine	mannose	0.025	0.047	0.1977	0.119	0.232	0.2333
creatinine	tyrosine	0.021	0.041	0.2030	0.004	0.016	0.4171
creatinine	histidine	< 0.001	< 0.001	0.4068	< 0.001	< 0.001	0.6272
creatinine	threonine	0.110	0.163	0.1416	0.014	0.045	0.3601
creatinine	lysine	< 0.001	< 0.001	0.5762	0.394	0.554	0.1288
creatinine	methionine	< 0.001	< 0.001	0.3395	< 0.001	< 0.001	0.5744
creatinine	unk1	0.004	0.010	0.2496	0.749	0.843	0.0485
creatinine	3-hydroxybutyrate	0.015	0.031	0.2137	0.338	0.499	-0.1445
creatinine	succinate	0.017	0.034	0.2097	0.096	0.193	-0.2486
creatinine	2-hydroxybutyrate	0.001	0.003	0.3008	0.909	0.958	-0.0174
creatinine	proline	0.001	0.003	0.2874	0.063	0.142	-0.2764

Supplementary Table 7. Multivariate statistical analysis of correlation between amino acids and other metabolites (DS, n=129; CTRL, n=46). The table contains the statistically significant correlations (in DS and/or CTRL group) between metabolites analyzed with 1H-NMR from plasma samples. We reported p-value of the univariate Wilcoxon test, p-value after FDR correction and r for each metabolite. Correlations with significant p-value after FDR correction (<0.01) are highlighted in red. Pairs with moderate correlation (0.4< r <0.7) are highlighted in green and pairs with strong correlation (r >0.7) are highlighted in blue.

Metabolite 1	Metabolite 2	DS Metabolites			CTRL Metabolites		
		P-value	P-value after FDR correction	r	P-value	P-value after FDR correction	r
alanine	leucine	0.001	0.003	0.2855	0.883	0.940	0.0222
alanine	isoleucine	0.002	0.005	0.2683	0.403	0.556	-0.1264
alanine	valine	< 0.001	< 0.001	0.3205	0.001	0.005	0.4785
alanine	pyruvate	0.006	0.014	0.2388	< 0.001	< 0.001	0.6332
alanine	acetone	< 0.001	< 0.001	-0.3213	0.086	0.179	-0.2561
alanine	glutamine	< 0.001	< 0.001	0.4360	< 0.001	< 0.001	0.5857
alanine	citrate	0.029	0.054	0.1923	0.016	0.049	0.3527
alanine	glycine	< 0.001	< 0.001	0.4637	< 0.001	< 0.001	0.6859
alanine	creatine	0.300	0.377	0.0919	< 0.001	< 0.001	-0.5290
alanine	creatinine	0.062	0.104	0.1646	0.004	0.016	0.4198
alanine	lactate	0.001	0.003	0.2794	< 0.001	< 0.001	0.5742
alanine	glucose	< 0.001	< 0.001	0.3946	< 0.001	< 0.001	0.6576
alanine	tyrosine	< 0.001	< 0.001	0.3150	< 0.001	< 0.001	0.6311
alanine	histidine	< 0.001	< 0.001	0.3333	< 0.001	< 0.001	0.6254
alanine	threonine	0.052	0.089	0.1718	0.006	0.022	0.3995
alanine	lysine	< 0.001	< 0.001	0.3332	0.069	0.150	-0.2703
alanine	acetatoacetate	< 0.001	< 0.001	-0.3134	0.027	0.075	-0.3260
alanine	methionine	0.808	0.856	-0.0216	0.001	0.005	0.4721
alanine	unk2	0.008	0.018	0.2319	0.211	0.350	0.1878
alanine	3-hydroxybutyrate	0.022	0.042	-0.2019	< 0.001	< 0.001	-0.5827
alanine	succinate	0.040	0.071	0.1814	< 0.001	< 0.001	-0.5245
alanine	2-hydroxybutyrate	0.670	0.738	-0.0378	0.007	0.025	-0.3894
alanine	proline	0.008	0.018	0.2310	< 0.001	< 0.001	-0.5671
glutamine	leucine	< 0.001	< 0.001	0.5966	0.005	0.019	0.4079
glutamine	isoleucine	< 0.001	< 0.001	0.4218	0.116	0.227	0.2352
glutamine	valine	< 0.001	< 0.001	0.5358	0.004	0.016	0.4156
glutamine	alanine	< 0.001	< 0.001	0.4360	< 0.001	< 0.001	0.5857
glutamine	acetate	< 0.001	< 0.001	0.3691	0.396	0.554	0.1283
glutamine	pyruvate	0.021	0.041	0.2030	0.001	0.005	0.4880
glutamine	citrate	< 0.001	< 0.001	0.4807	0.005	0.019	0.4052
glutamine	glycine	< 0.001	< 0.001	0.6634	< 0.001	< 0.001	0.6924
glutamine	creatine	< 0.001	< 0.001	0.3426	0.636	0.766	-0.0716
glutamine	creatinine	< 0.001	< 0.001	0.5017	< 0.001	< 0.001	0.6029
glutamine	lactate	0.598	0.678	-0.0469	0.001	0.005	0.4553
glutamine	glucose	< 0.001	< 0.001	0.6416	< 0.001	< 0.001	0.6545
glutamine	mannose	0.004	0.010	0.2526	0.084	0.177	0.2571
glutamine	tyrosine	< 0.001	< 0.001	0.4152	0.001	0.005	0.4859
glutamine	histidine	< 0.001	< 0.001	0.7363	< 0.001	< 0.001	0.7056
glutamine	phenylalanine	0.002	0.005	0.2729	0.030	0.081	0.3201
glutamine	fumarate	0.063	0.105	0.1643	0.004	0.016	0.4192
glutamine	threonine	0.001	0.003	0.2879	0.073	0.157	0.2665
glutamine	lysine	< 0.001	< 0.001	0.7336	0.039	0.099	0.3053
glutamine	methionine	0.001	0.003	0.2889	0.003	0.013	0.4290
glutamine	unk1	< 0.001	< 0.001	0.3382	0.225	0.365	0.1824
glutamine	unk3	< 0.001	< 0.001	0.3788	0.020	0.058	0.3422
glutamine	succinate	< 0.001	< 0.001	0.3457	0.766	0.856	-0.0451
glutamine	2-hydroxybutyrate	< 0.001	< 0.001	0.3273	0.963	0.986	-0.0070
glutamine	proline	< 0.001	< 0.001	0.3588	0.479	0.629	-0.1070
glycine	leucine	0.001	0.003	0.3017	0.323	0.484	0.1490
glycine	valine	0.003	0.008	0.2602	< 0.001	< 0.001	0.5527
glycine	alanine	< 0.001	< 0.001	0.4637	< 0.001	< 0.001	0.6859
glycine	pyruvate	0.001	0.003	0.2876	< 0.001	< 0.001	0.6366
glycine	glutamine	< 0.001	< 0.001	0.6634	< 0.001	< 0.001	0.6924
glycine	citrate	< 0.001	< 0.001	0.3514	0.005	0.019	0.4092
glycine	creatine	< 0.001	< 0.001	0.4132	0.033	0.085	-0.3149
glycine	creatinine	0.004	0.010	0.2549	< 0.001	< 0.001	0.5763
glycine	lactate	0.437	0.524	0.0690	< 0.001	< 0.001	0.5434
glycine	glucose	< 0.001	< 0.001	0.5412	< 0.001	< 0.001	0.7217
glycine	tyrosine	0.008	0.018	0.2332	< 0.001	< 0.001	0.5293
glycine	histidine	< 0.001	< 0.001	0.4110	< 0.001	< 0.001	0.6698
glycine	formate	0.006	0.014	0.2394	0.064	0.142	0.2753
glycine	fumarate	0.001	0.003	0.2776	0.217	0.357	0.1856

glycine	threonine	< 0.001	< 0.001	0.3386	0.002	0.009	0.4436
glycine	lysine	< 0.001	< 0.001	0.5458	0.584	0.722	-0.0828
glycine	methionine	0.001	0.003	0.2789	< 0.001	< 0.001	0.5105
glycine	unk1	0.014	0.029	0.2151	0.529	0.681	0.0952
glycine	unk3	0.002	0.005	0.2656	0.044	0.107	0.2979
glycine	succinate	< 0.001	< 0.001	0.3963	0.005	0.019	-0.4096
glycine	2-hydroxybutyrate	0.002	0.005	0.2674	0.897	0.950	0.0197
glycine	proline	0.003	0.008	0.2584	0.002	0.009	-0.4534
tyrosine	leucine	0.001	0.003	0.2819	0.029	0.079	0.3225
tyrosine	isoleucine	0.002	0.005	0.2718	0.461	0.616	0.1114
tyrosine	valine	0.001	0.003	0.2821	< 0.001	< 0.001	0.5451
tyrosine	alanine	< 0.001	< 0.001	0.3150	< 0.001	< 0.001	0.6311
tyrosine	acetate	0.012	0.025	0.2200	0.805	0.880	0.0374
tyrosine	pyruvate	0.362	0.444	-0.0808	0.002	0.009	0.4370
tyrosine	acetone	0.023	0.044	-0.1997	0.114	0.224	-0.2364
tyrosine	glutamine	< 0.001	< 0.001	0.4152	0.001	0.005	0.4859
tyrosine	glycine	0.008	0.018	0.2332	< 0.001	< 0.001	0.5293
tyrosine	creatinine	0.021	0.041	0.2030	0.004	0.016	0.4171
tyrosine	lactate	0.237	0.306	-0.1048	0.004	0.016	0.4178
tyrosine	glucose	< 0.001	< 0.001	0.3153	0.001	0.005	0.4614
tyrosine	mannose	0.005	0.012	0.2482	0.008	0.028	0.3888
tyrosine	histidine	0.001	0.003	0.3010	< 0.001	< 0.001	0.5507
tyrosine	threonine	0.019	0.038	0.2065	0.016	0.049	0.3539
tyrosine	lysine	< 0.001	< 0.001	0.3631	0.972	0.991	0.0054
tyrosine	acetatoacetate	0.194	0.260	-0.1151	0.076	0.162	-0.2646
tyrosine	methionine	0.054	0.092	0.1701	0.010	0.034	0.3777
tyrosine	3-hydroxybutyrate	0.203	0.267	-0.1129	0.001	0.005	-0.4644
tyrosine	succinate	0.799	0.849	0.0227	0.017	0.051	-0.3503
tyrosine	proline	0.075	0.119	0.1573	0.007	0.025	-0.3896
histidine	leucine	< 0.001	< 0.001	0.4725	0.048	0.114	0.293
histidine	isoleucine	< 0.001	< 0.001	0.4102	0.458	0.614	0.112
histidine	valine	< 0.001	< 0.001	0.4340	< 0.001	< 0.001	0.604
histidine	alanine	< 0.001	< 0.001	0.3333	< 0.001	< 0.001	0.625
histidine	acetate	0.004	0.010	0.2520	0.425	0.581	0.121
histidine	pyruvate	0.004	0.010	0.2537	< 0.001	< 0.001	0.644
histidine	glutamine	< 0.001	< 0.001	0.7363	< 0.001	< 0.001	0.706
histidine	citrate	< 0.001	< 0.001	0.4358	0.002	0.009	0.441
histidine	glycine	< 0.001	< 0.001	0.4110	< 0.001	< 0.001	0.670
histidine	creatine	< 0.001	< 0.001	0.3263	0.091	0.186	-0.252
histidine	creatinine	< 0.001	< 0.001	0.4068	< 0.001	< 0.001	0.627
histidine	lactate	0.530	0.618	0.0558	< 0.001	< 0.001	0.531
histidine	glucose	< 0.001	< 0.001	0.3812	< 0.001	< 0.001	0.614
histidine	tyrosine	0.001	0.003	0.3010	< 0.001	< 0.001	0.551
histidine	phenylalanine	0.020	0.039	0.2047	0.145	0.265	0.218
histidine	lysine	< 0.001	< 0.001	0.4892	0.917	0.958	-0.016
histidine	methionine	0.086	0.135	0.1516	0.002	0.009	0.437
histidine	unk1	0.004	0.010	0.2526	0.361	0.523	0.138
histidine	succinate	0.006	0.014	0.2410	0.019	0.056	-0.345
histidine	proline	0.008	0.018	0.2318	0.003	0.013	-0.427
phenylalanine	leucine	< 0.001	< 0.001	0.4165	0.126	0.240	0.2286
phenylalanine	isoleucine	< 0.001	< 0.001	0.4122	0.033	0.085	0.3158
phenylalanine	valine	< 0.001	< 0.001	0.4315	0.729	0.837	-0.0525
phenylalanine	glutamine	0.002	0.005	0.2729	0.030	0.081	0.3201
phenylalanine	glucose	0.004	0.010	0.2539	0.991	0.996	0.0017
phenylalanine	histidine	0.020	0.039	0.2047	0.145	0.265	0.2181
phenylalanine	lysine	< 0.001	< 0.001	0.3381	0.012	0.039	0.3683
phenylalanine	acetatoacetate	0.023	0.044	-0.1996	0.033	0.085	-0.3141
phenylalanine	unk3	0.183	0.248	0.1179	0.001	0.005	0.4924
threonine	isoleucine	0.011	0.023	0.2223	0.470	0.624	-0.1091
threonine	valine	0.025	0.047	0.1977	0.086	0.179	0.2561
threonine	alanine	0.052	0.089	0.1718	0.006	0.022	0.3995
threonine	acetate	0.013	0.027	0.2181	0.211	0.350	-0.1880
threonine	pyruvate	0.866	0.901	0.0150	0.007	0.025	0.3952
threonine	glutamine	0.001	0.003	0.2879	0.073	0.157	0.2665
threonine	glycine	< 0.001	< 0.001	0.3386	0.002	0.009	0.4436
threonine	creatinine	0.110	0.163	0.1416	0.014	0.045	0.3601
threonine	mannose	0.006	0.014	0.2422	0.085	0.178	0.2568
threonine	tyrosine	0.019	0.038	0.2065	0.016	0.049	0.3539
threonine	fumarate	0.018	0.036	0.2086	0.663	0.788	0.0659
threonine	lysine	0.004	0.010	0.2542	0.539	0.690	-0.0930

threonine	unk3	0.006	0.014	0.2414	0.623	0.756	0.0744
lysine	leucine	< 0.001	< 0.001	0.8479	< 0.001	< 0.001	0.649
lysine	isoleucine	< 0.001	< 0.001	0.6307	< 0.001	< 0.001	0.764
lysine	valine	< 0.001	< 0.001	0.8100	0.595	0.728	-0.081
lysine	alanine	< 0.001	< 0.001	0.3332	0.069	0.150	-0.270
lysine	acetate	< 0.001	< 0.001	0.3228	0.081	0.171	-0.260
lysine	pyruvate	< 0.001	< 0.001	0.3435	0.256	0.401	-0.171
lysine	glutamine	< 0.001	< 0.001	0.7336	0.039	0.099	0.305
lysine	citrate	< 0.001	< 0.001	0.3607	0.223	0.364	-0.183
lysine	glycine	< 0.001	< 0.001	0.5458	0.584	0.722	-0.083
lysine	creatine	< 0.001	< 0.001	0.5422	< 0.001	< 0.001	0.711
lysine	creatinine	< 0.001	< 0.001	0.5762	0.394	0.554	0.129
lysine	glucose	< 0.001	< 0.001	0.6239	0.069	0.150	-0.271
lysine	mannose	< 0.001	< 0.001	0.4136	0.333	0.493	-0.146
lysine	tyrosine	< 0.001	< 0.001	0.3631	0.972	0.991	0.005
lysine	histidine	< 0.001	< 0.001	0.4892	0.917	0.958	-0.016
lysine	phenylalanine	< 0.001	< 0.001	0.3381	0.012	0.039	0.368
lysine	formate	0.018	0.036	0.2078	0.403	0.556	-0.126
lysine	threonine	0.004	0.010	0.2542	0.539	0.690	-0.093
lysine	methionine	< 0.001	< 0.001	0.4765	0.139	0.256	-0.221
lysine	unk1	< 0.001	< 0.001	0.4463	0.091	0.186	0.252
lysine	unk3	< 0.001	< 0.001	0.3933	< 0.001	< 0.001	0.564
lysine	3-hydroxybutyrate	0.003	0.008	0.2628	< 0.001	< 0.001	0.590
lysine	succinate	0.002	0.005	0.2703	< 0.001	< 0.001	0.844
lysine	2-hydroxybutyrate	< 0.001	< 0.001	0.5244	0.346	0.507	0.142
lysine	proline	< 0.001	< 0.001	0.3643	< 0.001	< 0.001	0.835
methionine	leucine	< 0.001	< 0.001	0.3528	0.127	0.240	0.228
methionine	valine	< 0.001	< 0.001	0.3413	< 0.001	< 0.001	0.610
methionine	alanine	0.808	0.856	-0.0216	0.001	0.005	0.472
methionine	acetate	0.656	0.730	0.0396	0.010	0.034	0.376
methionine	pyruvate	0.016	0.032	0.2108	< 0.001	< 0.001	0.578
methionine	glutamine	0.001	0.003	0.2889	0.003	0.013	0.429
methionine	citrate	0.163	0.230	0.1234	< 0.001	< 0.001	0.509
methionine	glycine	0.001	0.003	0.2789	< 0.001	< 0.001	0.510
methionine	creatine	< 0.001	< 0.001	0.4404	0.054	0.126	-0.286
methionine	creatinine	< 0.001	< 0.001	0.3395	< 0.001	< 0.001	0.574
methionine	lactate	0.848	0.892	0.0171	< 0.001	< 0.001	0.557
methionine	glucose	< 0.001	< 0.001	0.3232	< 0.001	< 0.001	0.663
methionine	mannose	0.014	0.029	0.2158	0.075	0.161	0.265
methionine	tyrosine	0.054	0.092	0.1701	0.010	0.034	0.378
methionine	histidine	0.086	0.135	0.1516	0.002	0.009	0.437
methionine	formate	0.608	0.688	0.0456	0.005	0.019	0.409
methionine	lysine	< 0.001	< 0.001	0.4765	0.139	0.256	-0.221
methionine	acetatoacetate	0.012	0.025	0.2217	0.193	0.329	0.196
methionine	succinate	0.094	0.145	0.1479	< 0.001	< 0.001	-0.514
methionine	2-hydroxybutyrate	< 0.001	< 0.001	0.4144	0.292	0.448	0.159
methionine	proline	0.044	0.077	0.1779	< 0.001	< 0.001	-0.504
proline	leucine	< 0.001	< 0.001	0.3078	0.032	0.085	0.3170
proline	isoleucine	0.033	0.060	0.1878	< 0.001	< 0.001	0.5774
proline	valine	0.001	0.003	0.2879	0.001	0.005	-0.4883
proline	alanine	0.008	0.018	0.2310	< 0.001	< 0.001	-0.5671
proline	acetate	< 0.001	< 0.001	0.3043	0.093	0.189	-0.2503
proline	pyruvate	< 0.001	< 0.001	0.3281	< 0.001	< 0.001	-0.5362
proline	acetone	< 0.001	< 0.001	0.4827	0.933	0.966	-0.0127
proline	glutamine	< 0.001	< 0.001	0.3588	0.479	0.629	-0.1070
proline	citrate	0.003	0.008	0.2572	0.019	0.056	-0.3447
proline	glycine	0.003	0.008	0.2584	0.002	0.009	-0.4534
proline	creatine	0.064	0.106	0.1638	< 0.001	< 0.001	0.7311
proline	creatinine	0.001	0.003	0.2874	0.063	0.142	-0.2764
proline	lactate	0.001	0.003	0.2871	0.007	0.025	-0.3939
proline	glucose	< 0.001	< 0.001	0.3076	< 0.001	< 0.001	-0.6027
proline	mannose	0.253	0.323	0.1015	0.012	0.039	-0.3667
proline	tyrosine	0.075	0.119	0.1573	0.007	0.025	-0.3896
proline	histidine	0.008	0.018	0.2318	0.003	0.013	-0.4270
proline	lysine	< 0.001	< 0.001	0.3643	< 0.001	< 0.001	0.8349
proline	acetatoacetate	< 0.001	< 0.001	0.5202	0.680	0.798	0.0624
proline	methionine	0.044	0.077	0.1779	< 0.001	< 0.001	-0.5040
proline	unk2	0.008	0.018	0.2340	0.463	0.617	0.1109
proline	unk3	0.105	0.157	0.1434	0.016	0.049	0.3525
proline	3-hydroxybutyrate	< 0.001	< 0.001	0.5540	< 0.001	< 0.001	0.7047

proline	succinate	0.001	0.003	0.2894	< 0.001	< 0.001	0.9766
proline	2-hydroxybutyrate	0.001	0.003	0.2844	0.371	0.532	0.1351

Supplementary Table 8. Analysis of correlation between the metabolite levels identified by NMR analysis and the AE scores collected from DS patients subjected to Griffiths-III test (n=22). The table contains the statistically significant correlations between metabolites analyzed with 1H-NMR from plasma samples and the two main AE scores abtained by Griffiths-III test: Scale A and B AE scores from Griffiths III test (G A AE, and G B AE, respectively). We reported p-value and r for each metabolite.

METABOLITE	COGNITIVE TEST	P-value	r
leucine	G A AE	0.951	-0.0140
isoleucine	G A AE	0.991	-0.0020
valine	G A AE	0.581	-0.1280
alanine	G A AE	0.577	-0.1290
acetate	G A AE	0.590	-0.1250
pyruvate	G A AE	0.671	-0.0980
acetone	G A AE	0.525	0.1470
glutamine	G A AE	0.959	0.0120
citrate	G A AE	0.435	0.1800
glycine	G A AE	0.528	0.1460
creatine	G A AE	0.598	-0.1220
creatinine	G A AE	0.472	0.1660
lactate	G A AE	0.952	0.0140
glucose	G A AE	0.390	-0.1980
mannose	G A AE	0.466	0.1680
tyrosine	G A AE	0.193	-0.2960
histidine	G A AE	0.746	0.0750
phenylalanine	G A AE	0.542	-0.1410
formate	G A AE	0.882	0.0350
fumarate	G A AE	0.484	0.1620
threonine	G A AE	0.070	0.4030
lysine	G A AE	0.657	-0.1030
acetatoacetate	G A AE	0.512	0.1520
methionine	G A AE	0.381	-0.2010
unk1	G A AE	0.723	0.0820
unk2	G A AE	0.375	-0.2040
unk3	G A AE	0.630	-0.1120
3-hydroxybutyrate	G A AE	0.434	0.1800
succinate	G A AE	0.842	0.0460
2-hydroxybutyrate	G A AE	0.475	0.1650
proline	G A AE	0.821	0.0530

METABOLITE	COGNITIVE TEST	P-value	r
leucine	G B AE	0.791	0.0610
isoleucine	G B AE	0.501	0.1560
valine	G B AE	0.762	-0.0700
alanine	G B AE	0.867	-0.0390
acetate	G B AE	0.273	-0.2510
pyruvate	G B AE	0.540	0.1420
acetone	G B AE	0.193	0.2960
glutamine	G B AE	0.819	0.0530
citrate	G B AE	0.173	0.3090
glycine	G B AE	0.558	0.1350
creatine	G B AE	0.666	0.1000
creatinine	G B AE	0.106	0.3630
lactate	G B AE	0.557	0.1360
glucose	G B AE	0.216	-0.2820
mannose	G B AE	0.596	0.1230
tyrosine	G B AE	0.075	-0.3970
histidine	G B AE	0.305	0.2350
phenylalanine	G B AE	0.507	-0.1530
formate	G B AE	0.727	-0.0810
fumarate	G B AE	0.747	0.0750
threonine	G B AE	0.401	0.1930
lysine	G B AE	0.978	-0.0060
acetatoacetate	G B AE	0.266	0.2540
methionine	G B AE	0.850	0.0440
unk1	G B AE	0.665	0.1000
unk2	G B AE	0.837	-0.0480
unk3	G B AE	0.442	-0.1770
3-hydroxybutyrate	G B AE	0.196	0.2940
succinate	G B AE	0.715	0.0850
2-hydroxybutyrate	G B AE	0.439	0.1780
proline	G B AE	0.929	-0.0210

Supplementary Table 9. Analysis of correlations between the metabolite levels identified by NMR analysis and the AE scores collected from DS patients subjected to WPPSI test (n=39). The table contains the statistically significant correlations between metabolites analyzed with 1H-NMR from plasma samples and the three main AE scores abtained by WPPSI test: Verbal, Non Verbal and Total age equivalent scores from WPPSI test (W Verb AE, W Non Verb AE, W Tot AE, respectively). We reported p-value and r for each metabolite.

METABOLITE	COGNITIVE TEST	P-value	r
leucine	W Verb AE	0.302	0.1718
isoleucine	W Verb AE	0.649	-0.0764
valine	W Verb AE	0.341	0.1587
alanine	W Verb AE	0.921	-0.0167
acetate	W Verb AE	0.140	0.2439
pyruvate	W Verb AE	0.783	0.0463
acetone	W Verb AE	0.964	-0.0076
glutamine	W Verb AE	0.127	0.2518
citrate	W Verb AE	0.099	0.2714
glycine	W Verb AE	0.241	0.1950
creatine	W Verb AE	0.544	0.1015
creatinine	W Verb AE	0.130	0.2502
lactate	W Verb AE	0.909	0.0192
glucose	W Verb AE	0.291	0.1757
mannose	W Verb AE	0.099	0.2715
tyrosine	W Verb AE	0.813	0.0397
histidine	W Verb AE	0.399	0.1409
phenylalanine	W Verb AE	0.628	0.0812
formate	W Verb AE	0.126	0.2525
fumarate	W Verb AE	0.490	-0.1156
threonine	W Verb AE	0.229	0.1998
lysine	W Verb AE	0.168	0.2281
acetatoacetate	W Verb AE	0.909	0.0192
methionine	W Verb AE	0.421	-0.1343
unk1	W Verb AE	0.602	0.0874
unk2	W Verb AE	0.823	-0.0374
unk3	W Verb AE	0.738	0.0562
3-hydroxybutyrate	W Verb AE	0.724	0.0591
succinate	W Verb AE	0.464	0.1223
2-hydroxybutyrate	W Verb AE	0.171	0.2267
proline	W Verb AE	0.260	-0.1872

METABOLITE	COGNITIVE TEST	P-value	r
leucine	W Non Verb AE	0.395	0.1419
isoleucine	W Non Verb AE	0.794	0.0438
valine	W Non Verb AE	0.323	0.1646
alanine	W Non Verb AE	0.116	-0.2593
acetate	W Non Verb AE	0.314	0.1677
pyruvate	W Non Verb AE	0.228	0.2004
acetone	W Non Verb AE	0.224	0.2021
glutamine	W Non Verb AE	0.354	0.1547
citrate	W Non Verb AE	0.120	0.2566
glycine	W Non Verb AE	0.749	-0.0537
creatine	W Non Verb AE	0.501	0.1125
creatinine	W Non Verb AE	0.105	0.2672
lactate	W Non Verb AE	0.338	0.1597
glucose	W Non Verb AE	0.843	-0.0331
mannose	W Non Verb AE	0.968	-0.0068
tyrosine	W Non Verb AE	0.568	-0.0957
histidine	W Non Verb AE	0.760	0.0511
phenylalanine	W Non Verb AE	0.909	0.0191
formate	W Non Verb AE	0.729	0.0581
fumarate	W Non Verb AE	0.394	0.1423
threonine	W Non Verb AE	0.553	0.0994
lysine	W Non Verb AE	0.862	0.0293
acetatoacetate	W Non Verb AE	0.781	0.0467
methionine	W Non Verb AE	0.456	0.1246
unk1	W Non Verb AE	0.125	0.2532
unk2	W Non Verb AE	0.532	0.1047
unk3	W Non Verb AE	0.607	-0.0863
3-hydroxybutyrate	W Non Verb AE	0.702	0.0642
succinate	W Non Verb AE	0.264	0.1859
2-hydroxybutyrate	W Non Verb AE	0.240	0.1951
proline	W Non Verb AE	0.170	-0.2270

METABOLITE	COGNITIVE TEST	P-value	r
leucine	W Tot AE	0.256	0.1889
isoleucine	W Tot AE	0.812	-0.0400
valine	W Tot AE	0.258	0.1883
alanine	W Tot AE	0.475	-0.1194
acetate	W Tot AE	0.123	0.2543
pyruvate	W Tot AE	0.482	0.1177
acetone	W Tot AE	0.644	0.0774
glutamine	W Tot AE	0.122	0.2549
citrate	W Tot AE	0.057	0.3118
glycine	W Tot AE	0.451	0.1260
creatine	W Tot AE	0.460	0.1234
creatinine	W Tot AE	0.067	0.3000
lactate	W Tot AE	0.631	0.0804
glucose	W Tot AE	0.474	0.1198
mannose	W Tot AE	0.221	0.2034
tyrosine	W Tot AE	0.956	-0.0093
histidine	W Tot AE	0.444	0.1281
phenylalanine	W Tot AE	0.678	0.0695
formate	W Tot AE	0.193	0.2157
fumarate	W Tot AE	0.862	-0.0292
threonine	W Tot AE	0.247	0.1927
lysine	W Tot AE	0.265	0.1853
acetatoacetate	W Tot AE	0.840	0.0338
methionine	W Tot AE	0.763	-0.0507
unk1	W Tot AE	0.306	0.1707
unk2	W Tot AE	0.930	0.0147
unk3	W Tot AE	0.966	0.0071
3-hydroxybutyrate	W Tot AE	0.671	0.0713
succinate	W Tot AE	0.309	0.1694
2-hydroxybutyrate	W Tot AE	0.126	0.2525
proline	W Tot AE	0.154	-0.2357
